# Mutational dissection of HCMV gB and gH cytoplasmic tails highlights conserved and divergent features of fusion regulation

**DOI:** 10.1128/mbio.01905-25

**Published:** 2025-09-22

**Authors:** Chanyoung Lee, Hannah K. DiBella, Ekaterina E. Heldwein

**Affiliations:** 1 Department of Molecular Biology and Microbiology, Tufts University School of Medicine 12261, Boston, Massachusetts, USA; 2 Graduate Program in Molecular Microbiology, Graduate School of Biomedical Sciences, Tufts University School of Medicine, Boston, Massachusetts, USA; 3 Department of Biological and Biomedical Science, Rowan University 3536, Glassboro, New Jersey, USA; The University of North Carolina at Chapel Hill, Chapel Hill, North Carolina, USA

**Keywords:** human cytomegalovirus, HCMV, herpesvirus, glycoprotein B, glycoprotein H, cytoplasmic domains, cytoplasmic tails, split-luciferase assay, cell-cell fusion, activation, inhibition

## Abstract

In herpesviruses, the fusogenic activity of the conserved glycoproteins gB and gH is regulated by their cytoplasmic (or intraviral) tails. However, prior to this work, their regulatory mechanisms had only been investigated in *Alpha-* and *Gamma-* but not in *Betaherpesvirinae*. Here, we developed a plasmid-transfection-based split-luciferase assay as a quantitative platform for measuring cell-cell fusion mediated by glycoproteins from human cytomegalovirus (HCMV), a member of *Betaherpesvirinae*. Using this assay, we confirmed that glycoproteins gB, gH, gL, and gO, along with the PDGFRα receptor, are necessary and sufficient for efficient cell-cell fusion in two distinct HCMV strains, TR and AD169. To investigate the roles of the cytoplasmic tails of HCMV TR gB and gH in membrane fusion, we generated a series of truncation and point mutants analogous to those that have hyper- or hypo-fusogenic phenotypes in herpes simplex virus 1 (HSV-1). We found that, similarly to HSV-1, the C-terminal amphipathic helix in the HCMV gB cytoplasmic tail restricts fusion, whereas the entire HCMV gH cytoplasmic tail is required for fusion activation. However, the structure of the HCMV gB cytoplasmic tail and its interactions with the cytoplasmic tail of gH might be different from HSV-1. We hypothesize that while in HCMV—as in HSV-1—the cytoplasmic tails of HCMV gB and gH function as an inhibitory clamp and an activating wedge, their structures and interactions differ from HSV-1, implicating potential mechanistic differences in fusion regulation between herpesvirus subfamilies.

**IMPORTANCE:**

Herpesviruses promote membrane fusion during infection using a complex multi-component membrane fusion machinery. Proper spatiotemporal deployment of such machinery is subject to precise regulatory inputs. One of these is the regulation of the fusogenic activity of the conserved herpesviral glycoproteins gB and gH by their cytoplasmic (or intraviral) domains. This regulatory mechanism has been investigated in *Alpha-* and *Gamma-* but not yet in *Betaherpesvirinae*. Here, we combined mutagenesis targeting the cytoplasmic tails of HCMV gB and gH with a plasmid-transfection-based split-luciferase assay for measuring cell-cell fusion, which we developed here. By testing a panel of truncation mutants, we showed that inhibitory and activating regulatory regions in gB and gH, respectively, are conserved in HCMV. However, none of the single point mutants of gB had expected phenotypes, suggesting that the cytoplasmic tails of HCMV gB and gH have distinct structures and interactions. This study introduces a robust and sensitive *in vitro* cell-cell fusion assay for probing HCMV fusion mechanism and shows that despite sequence conservation, the cytoplasmic domains of gB and gH may regulate fusion by distinct mechanisms. This knowledge may inform future efforts in rational vaccine design and antiviral development.

## INTRODUCTION

Membrane fusion is a fundamental biological process that enables enveloped viruses to enter host cells by merging the viral envelope with the host membrane. This process is mediated by viral fusogens that refold from a metastable prefusion conformation to a stable postfusion conformation while interacting with the opposing membranes (reviewed in references [1, 2]). These major conformational changes are believed to provide the necessary energy for fusion. The activity of fusogens is tightly regulated to ensure proper spatiotemporal deployment.

*Herpesviridae—*a family of large double-stranded DNA viruses that infect mammals, birds, and reptiles—employ a particularly complex membrane fusion mechanism. Unlike most enveloped viruses that rely on a single fusogen for membrane merger (reviewed in references [1, 2]), herpesviruses utilize a set of surface glycoproteins. The key players in this process are glycoprotein B (gB) and the glycoprotein H/glycoprotein L (gH/gL) complex, which are highly conserved across the *Herpesviridae* family. gB is composed of an N-terminal ectodomain (ecto), a membrane-proximal region (MPR), a hydrophobic transmembrane domain (TMD), and an intraviral, C-terminal cytoplasmic domain (CTD). gB is a homotrimeric class III membrane fusogen (reviewed in references [1, 3]). Unlike many class III fusogens that are activated by exposure to low pH, gB is activated by the gH/gL complex (reviewed in references [4, 5]). The gH/gL complex is a heterodimer containing an N-terminal ectodomain (ecto), a transmembrane domain (TMD), and a short C-terminal cytotail (CT) (reviewed in reference [4]). gL lacks a TMD and co-folds with the gH N terminus (reviewed in reference [4]). Depending on a herpesvirus, gH/gL triggers gB activation either upon binding a host receptor directly or as a larger complex with additional viral proteins that bind host receptors, conferring host tropism (reviewed in reference [5]).

Human cytomegalovirus (HCMV) is a member of the *Betaherpesvirinae* subfamily of *Herpesviridae*. It is the primary cause of congenital abnormalities, such as hearing loss, blindness, epilepsy, and microcephaly, in approximately 1% of newborns (reviewed in reference [6]). In addition, HCMV can cause disease in immunocompromised individuals, such as solid organ transplant patients and patients with AIDS, leading to conditions such as gastrointestinal ulceration, hepatitis, pneumonitis, and retinitis (reviewed in reference [7]).

In HCMV, cell entry requires fusogen gB and two gH/gL complexes: gH/gL/gO (trimer) and gH/gL/UL128-131 (pentamer), which have distinct functions. The trimer is required for entry into all cell types, including fibroblasts, epithelial, endothelial, and myeloid cells, and binds platelet-derived growth factor receptor alpha (PDGFRα) (8). Along with gB, the trimer is also necessary for efficient cell-cell fusion (9) and efficient fusion during HCMV entry (10). By contrast, the pentamer is required for entry into epithelial and endothelial cells and binds neuropilin-2 (Nrp2) (reviewed in reference [11]).

While the ectodomains of gB and gH are directly involved in membrane fusion, their cytoplasmic (intraviral) tails have regulatory roles (12, 13). The cytoplasmic domain of gB (gB_CTD_) inhibits the fusogenic activity of gB because point mutations, truncations, or insertions in the gB_CTD_ increase the extent of cell-cell fusion (14–28). Such mutations are thus referred to as hyper-fusogenic. This phenomenon has been extensively studied in the *Alphaherpesvirinae* herpes simplex viruses 1 and 2 (HSV-1 and HSV-2) and in the *Gammaherpesvirinae* Epstein-Barr virus (EBV). Infection with HSV-1 bearing hyper-fusogenic mutations in the gB_CTD_ causes the formation of multinucleated cells (syncytia) (14, 17, 29, 30). Similarly, transient expression of mutant gB proteins, along with other core entry glycoproteins, in uninfected cells increases cell-cell fusion in HSV-1 (31, 32), HSV-2 (15, 19, 24), and EBV (27, 33, 34). Based on mutational, structural, and biophysical studies in HSV-1, gB_CTD_ has been proposed to form a membrane-dependent inhibitory clamp that stabilizes the gB ectodomain in its prefusion conformation and prevents its premature activation (12). By contrast, the cytoplasmic tail of gH (gH_CT_) has been proposed to have an activating role. Progressive truncations of gH_CT_ lead to a greater reduction in cell-cell fusion in HSV-1 (13, 25) and EBV (35). Finally, interaction between gB_CTD_ and gH_CT_ has been proposed to trigger the fusogenic refolding of gB (13).

To dissect the roles of the cytoplasmic domains in the HCMV homologs of gB and gH, here, we developed an HCMV-specific dual split-luciferase assay (SLA), adapted from an HSV-specific SLA (13, 36), which allowed real-time kinetic monitoring of fusion events. By combining the SLA with mutagenesis, we found that just as in HSV-1, the putative C-terminal amphipathic helix in HCMV gB_CTD_ has an inhibitory effect on fusion, whereas the entire HCMV gH_CT_ is indispensable for fusion and may have an activating role. Surprisingly, point mutations targeting conserved gB_CTD_ residues that have hyper- or hypo-fusogenic phenotypes in HSV failed to reproduce comparable phenotypes in HCMV. This suggests that while gB and gH cytoplasmic tails have analogous roles in HSV-1 and HCMV, their structures and interactions differ from HSV-1, implicating potential mechanistic differences in fusion regulation between herpesvirus subfamilies. Our study uncovers regulatory regions in HCMV entry glycoproteins and identifies phenotypic differences in cytoplasmic domain regulation between HCMV and HSV-1, laying the foundation for future studies of regulatory mechanisms controlling HCMV entry.

## RESULTS

### The optimization of the HSV-1-specific split-luciferase assay

Cell-cell fusion assays are commonly used as surrogate models to measure fusion mediated by herpesvirus entry glycoproteins. These range from microscopic counting of multinucleated cells (i.e., syncytia) (28, 37, 38) to detection of expression of a luciferase reporter (15, 27, 32, 34, 39). More recently, the split-luciferase assay, initially developed for studying HIV-mediated fusion (40), has been subsequently adapted to measure HSV fusion kinetics (36). This assay quantitatively measures cell-cell fusion over time between two distinct cell types: effector cells and target cells. Effector cells co-express viral entry glycoproteins (gD, gH, gL, gB) along with a portion of a split luciferase reporter (RLuc1-7), whereas target cells co-express the receptor and the complementary portion of the split luciferase reporter (RLuc8-11) (40). Upon cell fusion, the dual *Renilla* luciferase enzyme is reconstituted and produces a luminescent signal that allows for real-time measurement of fusion kinetics in intact cells with high sensitivity (36) (Fig. S1A).

The use of the split-luciferase assay (SLA) to measure cell-cell fusion mediated by HCMV entry glycoproteins has been reported (9). That study employed recombinant adenoviruses. Here, we developed an alternative, plasmid-transfection-based SLA by adapting the existing HSV-specific SLA protocol (13, 36). Given the reported low surface levels of HCMV glycoproteins, notably, gB (41, 42), and slower HCMV fusion kinetics (43) compared to HSV, we first optimized the SLA protocol for HSV-1 to enhance its absolute luminescence signal. These multiple optimization steps include the use of the fresh cell, single use of plasmids and EnduRen substrate aliquots, use of a plate reader with proper CO_2_ level, and seeding cells with proper confluency for optimal transfection. When effector and target cells were transfected as previously published (effector cells, gB:gH:gL:gD (BHLD):RLuc1−7 = 3:1:1:1:1; target cells, nectin-1:RLuc8−11 = 1:1) (13), we achieved an absolute luminescence signal of approximately 500,000 raw luminescence units (RLU) in the HSV-1-specific SLA at 8 hours post-co-cultivation, nearly doubling previously reported values (Fig. S1B and C). Importantly, despite this increase, the assay preserved the expected trend of higher fusion extent of the hyper-fusogenic HSV-1 mutant gB868, ~150% fusion extent compared to WT gB (Fig. S1D). These optimized conditions served as a foundation for adapting the SLA to HCMV, allowing for a more robust and sensitive analysis of HCMV glycoprotein-mediated fusion.

### The development of HCMV-specific SLA

**Fig 1:**
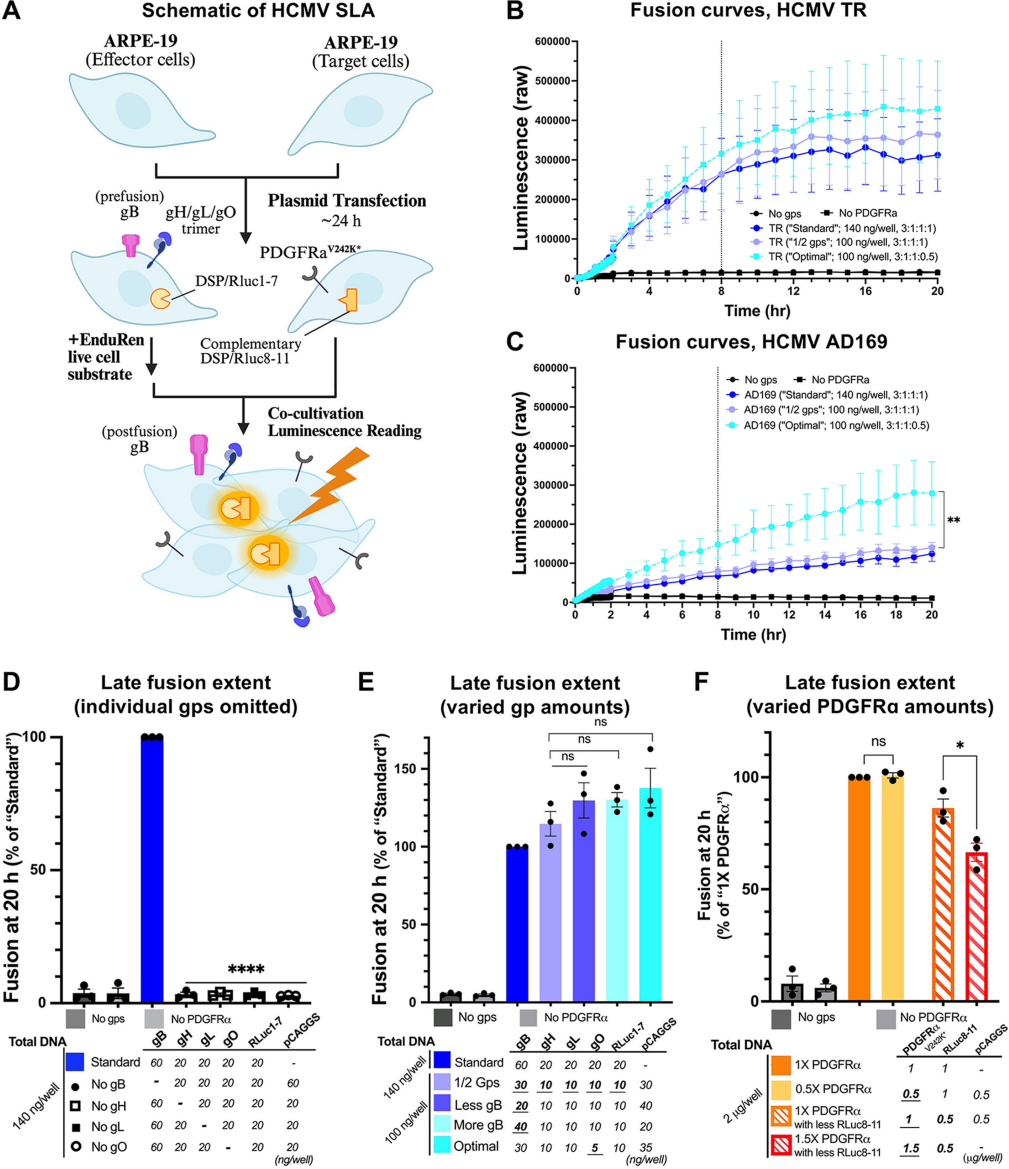
Development and optimization of a split-luciferase cell-cell fusion assay with HCMV entry glycoproteins. (**A**) Schematic representation of the experimental setup. ARPE-19 cells expressing HCMV gB, gH, gL, gO, and a fragment of *Renilla* luciferase (effector cells) are fusing with ARPE-19 cells expressing a mutated form of PDGFRα (PDGFRα^V242K*^) and a complementary fragment of *Renilla* luciferase (target cells). (**B and C**) Fusion curves of glycoproteins from HCMV strains TR (**B**) or AD169 (**C**). Raw luminescence values were plotted over 20 hours post-co-cultivation of effector and target cells. Each dot in curves represents a mean raw luminescence value from three biological replicates. Error bars represent SEM. (**D and F**) Late fusion extent in the absence of individual HCMV TR glycoproteins (**D**), with different amounts and ratios of glycoproteins (**E**), or different amounts of PDGFRα^V242K*^ (**F**). Late extent of fusion was defined as luminescence measured 20 hours post-co-cultivation of effector and target cells. The luminescence values in each group were normalized to the “Standard” condition. “No gps” and “No PDGFRɑ” served as negative controls. Bars represent the mean values from three biological replicates, each of which is the mean of three technical replicates. Error bars represent SEM. *P < 0.05, **P < 0.01, ***P < 0.001, ****P < 0.0001.

For the effector cells, we used human retinal pigment epithelial (ARPE-19) cells, which are susceptible to HCMV infection but have low endogenous expression of platelet-derived growth factor receptor alpha (PDGFRα) (44), which ensures low background fusion in the absence of target cells. HCMV entry glycoproteins gB, gH, gL, and gO from the HCMV TR or AD169 strain, along with RLuc1-7, were transiently expressed in the effector cells. The gH/gL/gO trimer was used because it is essential for HCMV entry across all cell types (8) and, along with gB, is necessary for efficient cell-cell fusion (9) and efficient fusion during HCMV entry (10) (Fig. 1A). Given broad genetic variation across HCMV glycoproteins, we chose two genetically distinct HCMV strains, the lab-adapted AD169 (reviewed in reference [45]) and the minimally passaged clinical isolate TR (46), which encode distinct gB, gH, and gO alleles (47, 48). To render target ARPE-19 cells more susceptible to fusion in the presence of gH/gL/gO, PDGFRα^V242K*^ was transiently expressed along with RLuc8-11 (PDGFRα^V242K*^:RLuc8−11 = 1:1) (Fig. 1A). PDGFRα^V242K*^ is a variant of human PDGFRα isoform 1 that contains a deletion of residues 1-23 and a V242K substitution. These modifications preserve the wild-type PDGFRα affinity for HCMV gH/gL/gO while abrogating binding to the native ligand PDGF (49), which increases the surface levels of PDGFRα by reducing its internalization in the presence of PDGF.

We defined as “Standard” the condition where the effector cells received a total of 140 ng/well of plasmid DNA at a ratio of gB:gH:gL:gO (BHLO):RLuc1−7 = 3:1:1:1:1 whereas target cells received a total of 2 µg/well of plasmid DNA at a ratio of PDGFRα^V242K*^:RLuc8−11 = 1:1. This yielded a strong luminescence signal with TR gB, gH, gL, and gO (Fig. 1B) and a somewhat weaker signal with the AD169 gB, gH, gL, and gO (Fig. 1C). The TR glycoproteins promoted more efficient fusion (Fig. 1B Vs. C) and were, therefore, used in subsequent experiments. Nonetheless, the SLA can be used to detect fusion mediated by glycoproteins from two genetically distinct strains, AD169 and TR.

To confirm that the cell-cell fusion in our system was dependent on HCMV glycoproteins and receptor, we conducted control experiments either in the absence of HCMV glycoproteins in effector cells (“No gps”) or in the absence of PDGFRα^V242K*^ in target cells (“No PDGFRα”). Both controls exhibited minimal fusion activity (less than 5% relative fusion extent) when normalized to the “Standard” condition (Fig. 1D). These two conditions were henceforth used as negative controls in all SLA experiments. In addition, we showed that each of the four glycoproteins—gB, gH, gL, and gO—was required for cell-cell fusion and that in the absence of any one of these, fusion activity was minimal (less than 5% relative fusion extent) (Fig. 1D).

To optimize fusion extent, we modified transfection conditions for effector and target cells. First, transfecting only half the DNA amount for HCMV glycoproteins and reducing the total amount of transfected DNA from 140 ng/well to 100 ng/well in effector cells while maintaining the same relative glycoprotein ratios did not significantly change the overall extent of fusion (“1/2 gps”; total 100 ng/well, BHLO = 3:1:1:1) (Fig. 1B, C and E). This is consistent with a prior HCMV SLA study (9). Next, we examined variations in glycoprotein ratios by testing “Less gB”: BHLO = 2:1:1:1; “More gB”: BHLO = 4:1:1:1; or “Less gO”; BHLO = 3:1:1:0.5, while keeping the total DNA amount constant at 100 ng/well. Varying the relative amount of gB had little effect on fusion extent (Fig. 1E). However, reducing the amount of gO slightly increased the fusion extent (Fig. 1B and E). In addition, this condition induced a significant increase in absolute fusion curves in the AD169 strain compared to the “1/2 gps” condition (Fig. 1C), suggesting that excessive gO expression may inhibit fusion. This effect could be attributed to gO being a substrate of ER-associated degradation (ERAD) (11). Therefore, we defined as “Optimal” the condition where the effector cells received reduced gO amount (“Less gO”: BHLO = 3:1:1:0.5).

Reducing the amount of PDGFRα^V242K*^ (“0.5X PDGFRα”) had little effect on fusion extent, whereas increasing the amount of PDGFRα^V242K*^ (“1.5X PDGFRα”) decreased fusion in a statistically significant manner (Fig. 1F). The observed reduction in fusion could be due to the difficulty in detaching cells following overexpression of PDGFRα^V242K*^ even after a prolonged treatment with a chelating agent. In the final, optimized protocol used in all subsequent SLA experiments, the effector cells were transfected with 100 ng/well of total DNA (BHLO = 3:1:1:0.5), whereas the target cells were transfected with 2 µg/well of total DNA (PDGFRα^V242K*^:RLuc8−11 = 1:1).

Relative to HSV-1 (Fig. S1B), fusion mediated by HCMV glycoproteins had a lower extent (Fig. 1B and C) and reduced early and late fusion rates (Fig. S1E and F). Hence, we chose 8 hours and 20 hours as early and late time points, respectively, for measurement of HCMV fusion extents and rates. In addition, HCMV glycoproteins delayed the initiation of fusion around 40 min, whereas HSV-1 glycoproteins initiated the fusion around 10 min (Fig. S1G). As fusion mediated by HCMV glycoproteins started post 40–60 min of co-cultivation of effector and target cells, we defined the early rate of fusion as the slope of the fusion curve between 1 and 8 hours post-co-cultivation of effector and target cells in HCMV SLA.

### Similarly to HSV-1 gB_CTD_, the putative helix h3 in HCMV gB_CTD_ has an inhibitory role whereas the rest of the gB_CTD_ is essential for fusion

**Fig 2:**
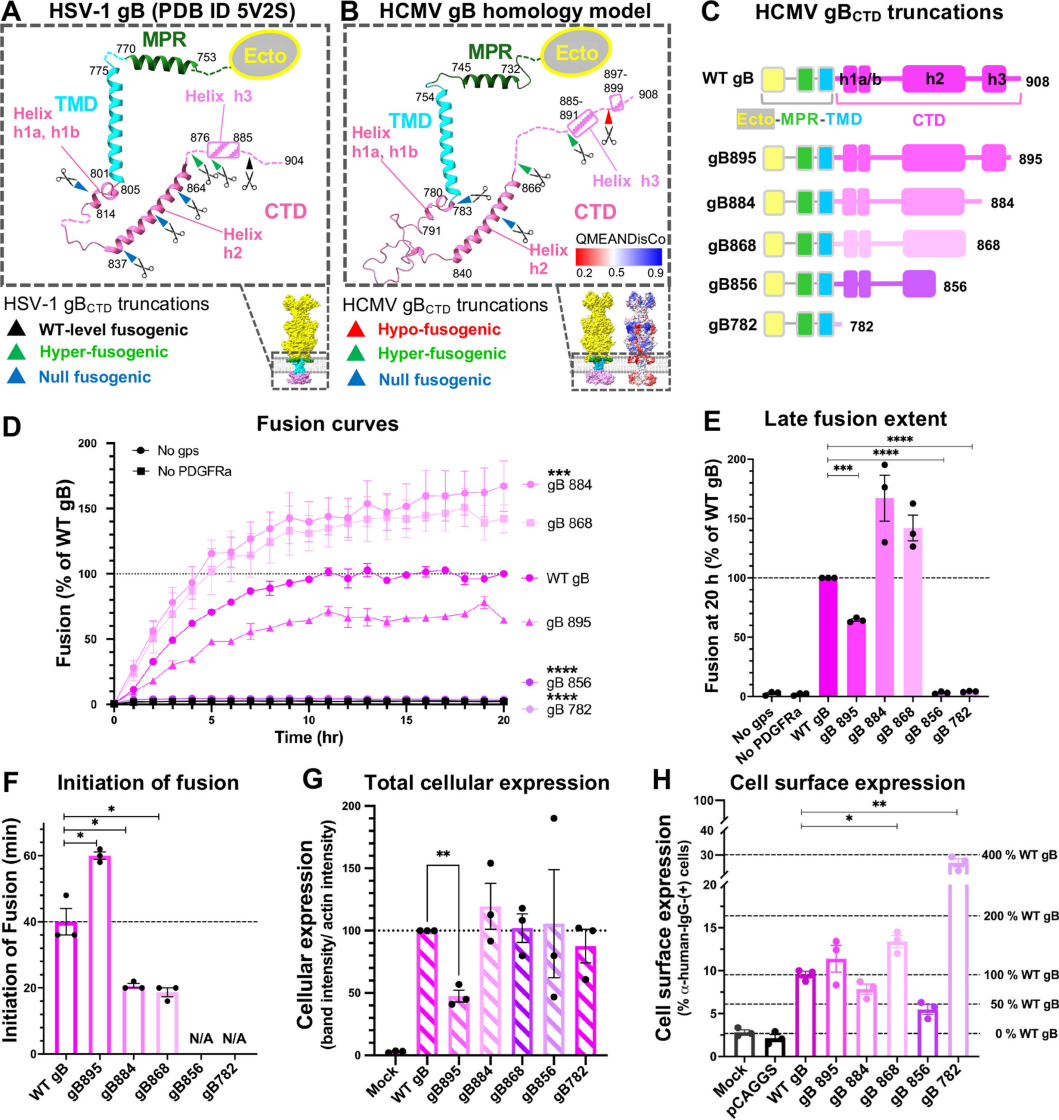
The putative amphipathic helix in the HCMV gB_CTD_ restricts fusion. (**A and B**) The secondary structure of the MPR-TMD-CTD region in a protomer from HSV-1 KOS gB crystal structure (PDB ID 5V2S) (**A**) or HCMV TR gB SWISS homology model (**B**). Boundaries of individual structural elements are labeled. Unresolved regions are indicated with dashed lines. In (**B**), modeling accuracy is represented by QMEANDisCO (Q mean distance constraint), with higher scores indicating higher confidence. Truncation sites in HSV-1 and HSV-2 gB_CTD_ mutants from previous studies (**A**) and of HCMV gB_CTD_ mutants in this study (**B**) are indicated with scissors, and their fusogenic phenotypes are indicated with arrows of different colors. (**C**) HCMV gB_CTD_ truncations tested in this study. (**D**) Fusion curves and (**E**) late fusion extent of the HCMV gB_CTD_ truncation mutants. Raw luminescence values were normalized to that of WT gB after 20 hours post-co-culture of effector and target cells. “No gps” and “No PDGFRɑ” served as negative controls. (**F**) Initiation of fusion of gB_CTD_ truncation mutants, defined as the first time point, where luminescence reached the value two-fold above the “No gps” control. Mutants that did not initiate the fusion within 20 hours are marked as N/A. (**G**) Total cellular expression of the gB_CTD_ truncation mutants in ARPE-19 cells was quantified from western blots in Fig. S2A. Cellular expression was first normalized to actin and then to WT gB. (**H**) Cell surface expression levels of the HCMV WT and mutant gB in ARPE-19 cells were measured by flow cytometry using anti-HCMV-gB human mAb 1G2 as a primary and Alexa488-conjugated goat anti-human mAb as a secondary antibody. In G-H, ARPE-19 cells either mock-transfected (“Mock”) or transfected with an empty vector (“pCAGGS”) served as negative controls. In (**D–H**), bars represent the mean values from three biological replicates with error bars representing SEM. *P < 0.05, **P < 0.01, ***P < 0.001, ****P < 0.0001.

The HSV-1 gB_CTD_ is composed of helices h1a, h1b, and h2 that form the folded trimeric core, resolved in the crystals of the nearly full-length HSV-1 gB (12) (Fig. 2A). An additional C-terminal amphipathic helix h3, unresolved in the crystal structure, was shown to interact with membrane by using electron spin resonance (12) (Fig. 2A). Both the C terminus of helix h2 and helix h3 are important inhibitory elements in gB homologs from HSV (15, 19, 24, 31, 32), PRV (50), and EBV gB (27, 33, 34), because their removal increases fusion. Larger C-terminal truncations of gB_CTD_ that remove portions of the pedestal reduce gB surface levels and abolish cell-cell fusion in HSV (18, 19, 30, 32) and EBV (27, 33, 34), and impaired ability to complement gB-null mutant in PRV (50) is likely due to protein misfolding.

To investigate the role of HCMV gB_CTD_ in fusion and identify regulatory elements, we first generated an *in silico* model of HCMV gB using SWISS-MODEL (Fig. 2B). SWISS-MODEL predicted four helices within the gB_CTD_, h1a, h1b, h2, and h3 (Fig. 2B), in accordance with the crystal structure of the HSV-1 gB (12) (Fig. 2A). To test the roles of the individual helices within HCMV gB_CTD_, we designed five gB_CTD_ mutants with partial or complete C-terminal truncations of predicted helices (Fig. 2C).

To assess their fusogenic potential, we measured the late fusion extent (20 hours) for each gB_CTD_ truncation mutant. Truncation of the unstructured C terminus (gB895) reduced fusion extent to ~70% (Fig. 2D and E). Larger truncations that eliminated h3 (gB884) or the unstructured linker between h2 and h3 (gB868) enhanced fusion extent to ~170% and ~140% of WT gB, respectively (Fig. 2D and E). These two truncations also induced earlier fusion initiation (Fig. 2F). Truncations that eliminated the C-terminal portion of h2 (gB856) or the entire CTD (gB782) reduced fusion to background levels (Fig. 2D and E). In addition, these two truncations prevented fusion initiation within 20 hours (Fig. 2F).

To rule out defects in mutant gB expression, we assessed total cellular expression levels in ARPE-19 cells using Western blot (WB) (Fig. 2G; Fig. S2A). The four shorter gB_CTD_ mutants (gB884, gB868, gB856, and gB782) were expressed at total levels comparable to WT gB (Fig. 2G), whereas the longer gB895 was expressed at a ~50% relative to WT gB (Fig. 2G; Fig. S2A). To check cell surface localization, we then measured surface expression levels by flow cytometry with mAb 1G2, which recognizes a conformational epitope in antigenic domain 5 (AD-5) of the gB ectodomain (Fig. 2H; Fig. S2B) (51). Mutations within the CTD are unlikely to affect the binding of 1G2 because its epitope retains its conformation in structures in both pre- and post-fusion forms of gB (52–55). The surface levels of WT HCMV gB and the four longer gB_CTD_ mutants (gB895, gB884, gB868, and gB856) were low (~5%–14% fluorescence-positive cells) (Fig. 2H; Fig. S2B) relative to HSV-1 WT gB (~70% fluorescence-positive cells) (Fig. S2C). However, the shortest mutant, gB782, which lacks the entire CTD, had significantly higher surface level, ~30% (Fig. 2H; Fig. S2B), likely due to the loss of a predicted tyrosine-based endocytic motif within helix h2 (YQML; residues 847–850), identified via Eukaryotic Linear Motif (ELM) analysis (http://elm.eu.org/search.html). Other studies have also reported low surface expression levels of HCMV gB (41, 42), presumably due to efficient endocytosis. Deletion of the entire HCMV gB_CTD_ led to a substantial increase in cell surface expression (41, 42). Analogous observations have been made for EBV gB (56, 57).

The reason for the reduced fusogenicity of the gB895 mutant (Fig. 2E) was unclear because its surface expression levels were comparable to WT gB (Fig. 2H). It could, perhaps, be less stable, which could also explain a 50% reduction in total cellular expression (Fig. 2G). The hyper-fusogenic phenotype of gB884 mutant (Fig. 2E) was not due to higher surface expression (Fig. 2H). Although the increased fusogenicity of gB868 could be partially due to higher surface expression (Fig. 2H), gB868 exhibited an earlier fusion initiation that was comparable to gB884 (Fig. 2F). Therefore, both gB868 and gB884 had hyper-fusogenic phenotypes. These results demonstrate that the putative amphipathic helix h3 in HCMV gB_CTD_ has an inhibitory role. Lastly, the fusion-null mutant gB856 was expressed at a reduced surface level yet above background, whereas the other fusion-null mutant gB782 was expressed at an increased level relative to WT gB (Fig. 2H). Since fusion-null phenotypes of these two mutants are not due to the lack of cell surface expression, we conclude that the HCMV gB_CTD_, especially the putative helix h2, is essential for fusion.

### Mutations of residues that have key regulatory roles in HSV-1 gB_CTD_ have divergent effects on function and cell surface expression in HCMV gB

To identify additional regulatory residues within gB_CTD_, we mapped previously characterized HSV-1 or HSV-2 gB_CTD_ point mutations associated with hyper-fusogenic (summarized in reference [12]); hypo-fusogenic, that is, reduced fusion (13); or low-surface level (15, 32) phenotypes onto the sequence alignments of gB_CTD_ from HSV-1 KOS and HCMV TR strains (Fig. S3).

**Fig 3:**
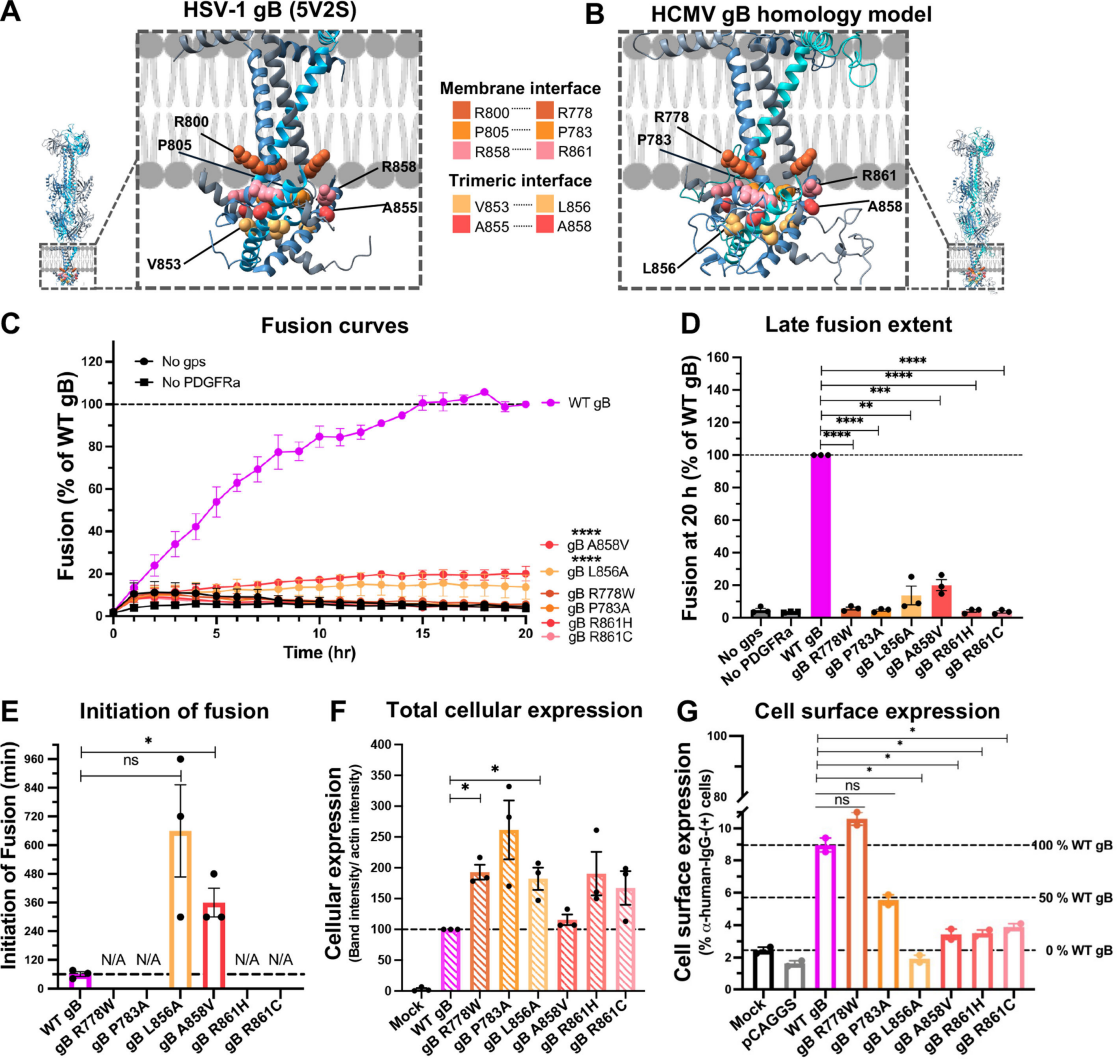
Single point mutants of conserved interface residues in HCMV gB_CTD_ have unexpected phenotypes. (**A**) Five functionally important conserved gB_CTD_ residues are located at either protein/membrane or trimeric interface in the HSV-1 gB crystal structure (PDB ID 5V2S). Their mutations cause a hyper-fusogenic phenotype. (**B**) The counterparts of residues in (**A**) in the HCMV TR gB homology model (SWISS homology modeling) targeted by mutagenesis. (**C**) Fusion curves and (**D**) late fusion extent of the HCMV gB_CTD_ interface mutants. Raw luminescence values were normalized to that of WT gB after 20 hours post-co-culture of effector and target cells. “No gps” and “No PDGFRɑ” served as negative controls. (**E**) Initiation of fusion of gB_CTD_ interface mutants. Mutants that did not initiate the fusion within 20 hours are marked as N/A. (**F**) Total cellular expression of the gB_CTD_ interface mutants in ARPE-19 cells was quantified from western blots shown in Fig. S4A. Cellular expression was first normalized to actin and then to WT gB. Mock-transfected cells (“Mock”) served as a negative control. (**G**) Cell surface expression levels of the HCMV gB_CTD_ interface mutants in ARPE-19 cells were measured by flow cytometry using anti-HCMV-gB human mAb 1G2 as a primary and Alexa488-conjugated goat anti-human mAb as a secondary antibody. ARPE-19 cells mock transfected (“Mock”) or transfected with an empty vector (“pCAGGS”) served as negative controls. Representative flow cytometry scatter plots are shown in Fig. S4C. In (**C–G**), bars represent the mean values from three biological replicates with error bars representing SEM. *P < 0.05, **P < 0.01, ***P < 0.001, ****P < 0.0001.

Point mutations targeting residues at protein/membrane or trimeric interfaces within the gB_CTD_ core in HSV-1 and HSV-2 are associated with hyper-fusogenic phenotypes (summarized in reference [12]). Five such residues in HSV-1 gB (R800, P805, V853, A855, and R858) (Fig. 3A) are conserved in HCMV gB (R778, P783, L856, A858, and R861) (Fig. S3) and also localize to the protein/membrane interface (R778, P783, and R861) or trimeric interfaces (L856 and A858) in the HCMV gB_CTD_ homology model (Fig. 3B). We generated six single-point mutants in HCMV gB_CTD_ by altering side chain size and/or polarity (gB R778W, P783A, L856A, A858V, R861H, and R861C). Unexpectedly, instead of hyper-fusogenic phenotypes, HCMV gB_CTD_ single-point mutants had very low fusion levels, <20% of WT gB (gB L856A and A858V) or background (gB R778W, P783A, R861H, and R861C) (Fig. 3C and D). Whereas gB L856A and A858V initiated the fusion much later than the WT gB, after 10 or 6 hours post-co-cultivation, respectively, the rest showed no fusion within 20 hours (Fig. 3E). None of the single-point mutants showed any defect in total cellular expression (Fig. 3F; Fig. S4A). However, only gB R778W and P783A were expressed on the cell surface at the above-background levels (Fig. 3G; Fig. S4C). Based on these results, we classified gB R778W and P783A mutants as hypo-fusogenic and gB L856A, A858V, R861H, and R861C mutants as low-surface level. These results indicate that, despite sequence conservation, some residues might not be functionally conserved, whereas others might, instead, be important for protein folding.

### The functional pocket of HSV-1 gB_CTD_ is not conserved in HCMV gB_CTD_

**Fig 4:**
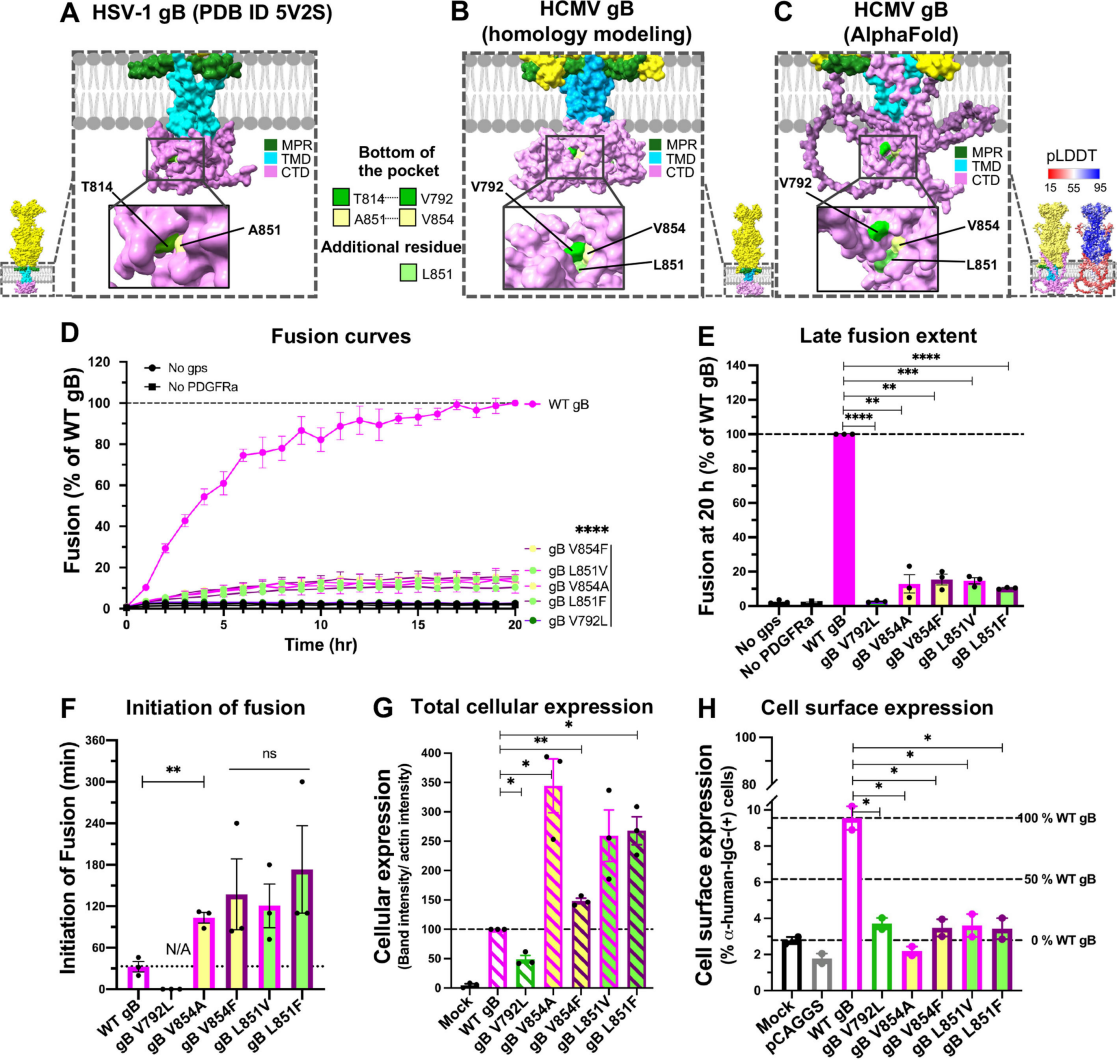
Single point mutants of residues in a putative surface pocket in HCMV gB_CTD_ cause protein misfolding. (**A**) Functionally important surface pocket in gB_CTD_ in HSV-1 gB crystal structure (PDB ID 5V2S). (**B and C**) The putative counterparts of the HCMV gB_CTD_ surface pocket in the Swiss homology model (**B**) and an AlphaFold model (**C**) targeted by mutagenesis. (**D**) Fusion curves and (**E**) late fusion extent of the HCMV gB_CTD_ pocket mutants. Raw luminescence values were normalized to that of WT gB after 20 hours post-co-culture of effector and target cells. “No gps” and “No PDGFRɑ” served as negative controls. (**F**) Initiation of fusion of gB_CTD_ pocket mutants. Mutants that did not initiate the fusion within 20 hours are marked as N/A. (**G**) Total cellular expression of the gB_CTD_ pocket mutants in ARPE-19 cells was quantified from western blots shown in Fig. S4B. Relative cellular expression was first normalized to actin and then to WT gB. Mock-transfected cells (“Mock”) served as a negative control. (**H**) Cell surface expression levels of the HCMV gB_CTD_ pocket mutants in ARPE-19 cells were measured by flow cytometry using anti-HCMV-gB human mAb 1G2 as a primary and Alexa488-conjugated goat anti-human mAb as a secondary antibody. ARPE-19 cells mock transfected (“Mock”) or transfected with an empty vector (“pCAGGS”) served as negative controls. Representative flow cytometry scatter plots are shown in Fig. S4D. In (**D–H**), bars represent the mean values from three biological replicates with error bars representing SEM. *P < 0.05, **P < 0.01, ***P < 0.001, ****P < 0.0001.

In HSV-1 gB_CTD_, T814L and A851V mutants have a rare hypo-fusogenic phenotype (13). Within the HSV-1 gB_CTD_ structure (12), T814 and A851 are positioned at the bottom of a surface-exposed pocket (Fig. 4A), which has been proposed to bind the gH_CT_ (13) according to the “clamp-and-wedge” model of gB activation by gH (12, 13).

Analogous pockets in structural models of HCMV gB_CTD_ generated by SWISS-MODEL (Fig. 4B) and AlphaFold (Fig. 4C) are lined by residues V792, L851, and V854. V792 and V854 in HCMV gB_CTD_ align with T814 and A851 in HSV-1 gB_CTD_ in sequence alignments (Fig. S3). To probe the role of the pocket residues, we generated five pocket-filling (V792L, L851F, and V854F) or pocket-emptying (L851V and V854A) mutants.

All mutants targeting residues L851 or V854 of HCMV gB_CTD_ had very low fusion levels, <20% of WT gB (Fig. 4D and E) and initiated fusion later than the WT gB, after 2–3 hours post-co-cultivation (Fig. 4F). These mutants showed no defect in total cellular expression (Fig. 4G; Fig. S4B) but had significantly reduced surface expression, comparable to background level (Fig. 4H; Fig. S4D). Therefore, we classified them as low-surface level. By contrast, gB V792L had background fusion levels (Fig. 4D and E) and had very low total cellular expression levels (Fig. 4G). These results show that despite sequence conservation, these residues in HCMV gB might be important for protein folding rather than function. Collectively, functional phenotypes of the mutations targeting putative interface and pocket residues point to fundamental differences in the predicted structures and/or regulatory mechanisms between HSV and HCMV.

### HCMV gH_CT_ is required for cell-cell fusion

**Fig 5:**
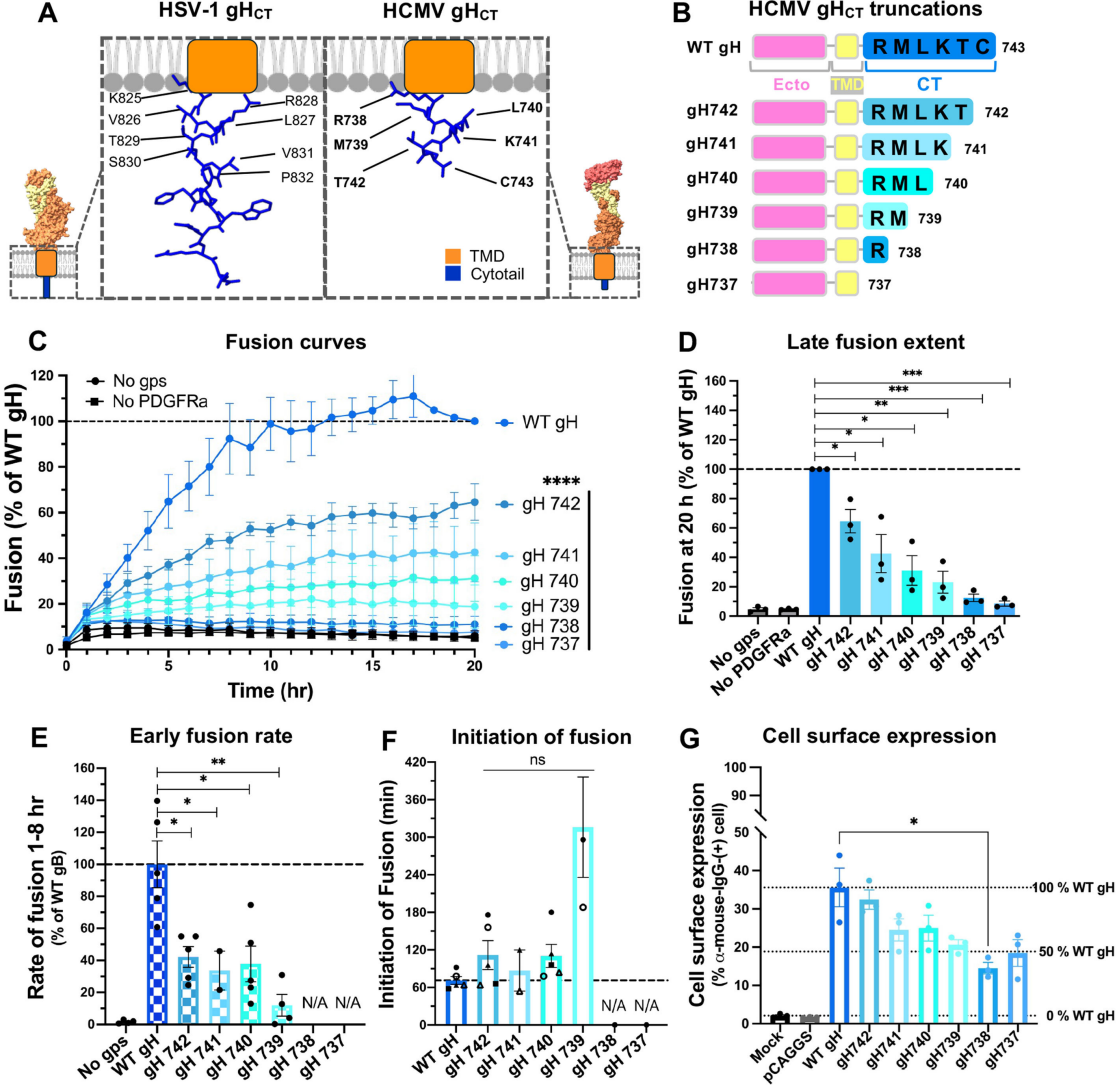
gH_CT_ is important for HCMV-mediated cell-cell fusion. (**A**) Models of gH_CT_ in HSV-1 (left) and HCMV (right). The eight residues of HSV-1 gH_CT_ that are important for fusion and six residues of HCMV gH_CT_ are labeled. (**B**) HCMV gH_CT_ truncations were tested in this study. (**C**) Fusion curves and (**D**) late fusion extent of the HCMV gH_CT_ truncation mutants. Raw luminescence values were normalized to that of WT gH after 20 hours post-co-culture of effector and target cells. “No gps” and “No PDGFRɑ” served as negative controls. (**E**) Early fusion rate, defined as the slope of the fusion curve between 1 and 8 hours post-co-culture. “No gps” served as a negative control. N/A: not applicable due to low values. (**F**) Initiation of fusion of the HCMV gH_CT_ truncation mutants. Mutants that did not initiate the fusion within 20 hours are marked as N/A. (**G**) Cell surface expression levels of the HCMV gH_CT_ truncation mutants in ARPE-19 cells were measured by flow cytometry using anti-HCMV-gB mouse mAb 14-4b as a primary and Alexa488-conjugated goat anti-mouse mAb as a secondary antibody. ARPE-19 cells mock transfected (“Mock”) or transfected with an empty vector (“pCAGGS”) served as negative controls. Representative flow cytometry scatter plots are shown in Fig. S6A. In (**C–G**), bars represent the mean values from three biological replicates with error bars representing SEM. *P < 0.05, **P < 0.01, ***P < 0.001, ****P < 0.0001.

In HSV-1, fusion requires not only the gH/gL ectodomain but also the 14-residue gH cytotail (gH_CT_) (Fig. 5A**,** left) (25). Truncation studies revealed that an eight-residue gH_CT_ was sufficient to maintain wild-type fusion levels, whereas further truncations led to a progressive decline in fusion, culminating in a full loss of function upon complete deletion (13).

HCMV gH_CT_ is significantly shorter across both clinical and laboratory-adapted strains, consisting of only six highly conserved residues (Fig. 5A**,** right and Fig. S5). To test its role in fusion regulation, we designed a panel of truncation mutants (gH742, gH741, gH740, gH739, gH738, and gH737) (Fig. 5B). Fusion efficiency decreased proportionally with the length of the remaining gH_CT_, culminating in a complete loss of fusion in gH737 (Fig. 5C and D), similarly to HSV-1 gH_CT_. In terms of fusion kinetics, gH742, gH741, and gH740 exhibited 40% of the early rate of fusion (Fig. 5E) and WT-level initiation of fusion (Fig. 5F). However, gH739 initiated fusion only after ~5 hours post co-cultivation (Fig. 5F), whereas gH738 and gH737 showed no fusion even after 20 hours (Fig. 5F).

To rule out defects in surface expression of the gH_CT_ truncations, we performed flow cytometry analysis, which showed that, unlike HCMV gB, HCMV gH consistently displayed high surface expression levels (Fig. 5G; Fig. S6A). Notably, a progressive reduction in surface expression was observed across the gH_CT_ truncations (Fig. 5G ; Fig. S6A), which is different from HSV-1 gH_CT_, in which truncations had no effect on cell surface expression (13). Reduction in cell surface expression did not account for the decrease in fusion, however, as can be seen from the progressive reduction in relative fusogenicity (Fig. S6B). Thus, efficient fusion requires all six gH_CT_ residues.

## DISCUSSION

In herpesviruses, the cytoplasmic (or intraviral) tails of the conserved glycoproteins gB and gH regulate their fusogenic activity. While this regulatory role has been characterized in *Alpha-* and *Gammaherpesvirinae*, it had not been investigated in *Betaherpesvirinae* prior to this study. Here, we generated a series of truncation and single point mutants in HCMV gB and gH cytoplasmic tails analogous to those that have hyper- or hypo-fusogenic phenotypes in HSV-1. By measuring the effect of mutations on cell-cell fusion using a plasmid-transfection-based split-luciferase assay, we showed that HCMV gB and gH have inhibitory and activating regulatory regions, respectively, that are conserved in other herpesviruses. We also uncovered phenotypic differences between analogous gB_CTD_ mutants in HCMV and HSV-1, likely due to structural differences.

The first question addressed in this study was whether the HCMV gB_CTD_ was as important for fusion and whether it restricted fusion. In HSV-1, the gB_CTD_ has been proposed to form a membrane-dependent inhibitory clamp that stabilizes the gB ectodomain in its prefusion conformation (12). This clamp is composed of the folded trimeric core, resolved in the crystals of the nearly full-length HSV-1 gB, and the C-terminal amphipathic helix that interacts with the membrane (12). Point mutations targeting protein/protein or protein/membrane interfaces within the gB_CTD_ core cause hyperfusogenic phenotypes in HSV-1 and HSV-2 (summarized in reference [12]). Truncations of the amphipathic helix cause hyperfusogenic phenotypes in HSV-1 (30–32), HSV-2 (19, 24), EBV (27, 33, 34), and larger plaques in PRV (50).

We found that truncations of the putative amphipathic helix h3 at the HCMV gB C terminus caused a hyperfusogenic phenotype (Fig. 2E), similarly to what has been observed in HSV-1 (30–32), HSV-2 (19, 24), PRV (50), and EBV (27, 33, 34). In HSV-1 gB, this helix has been shown to interact with the membrane and is proposed to act as a stabilizing membrane bilayer anchor for the inhibitory gB_CTD_ clamp (12). Larger truncations that eliminated a portion of helix h2 or the entire gB_CTD_ (gB856 and gB782, respectively) completely abolished fusion activity (Fig. 2E), suggesting that HCMV gB_CTD_, especially the putative helix h2, is essential for fusion. Surprisingly, removal of the last 13 residues (gB895) reduced fusion (Fig. 2E), which could be due to problems in protein synthesis and/or stability, as suggested by a reduced total expression (Fig. 2G). This contrasts with analogous deletions in HSV and EBV that retained wild-type fusion levels (19, 31). Taken together, these results show for the first time that HCMV gB_CTD_ is not only important for membrane fusion induced by HCMV glycoproteins but that its putative C-terminal amphipathic helix has an inhibitory role.

The second question addressed in this study was whether the HCMV gH_CT_ was as important for fusion. In HSV-1, the first 8 residues of the 14-residue gH_CT_ are essential for membrane fusion (13, 25, 58, 59). Truncations of HSV-1 gH_CT_ reduce cell-cell fusion, with fusion levels being proportionate to the length of the remaining gH_CT_ (13, 25). In EBV, the 8-residue gH_CT_ is also important for membrane fusion (35). Here, we found that the entire 6-residue HCMV gH_CT_ is important for cell-cell fusion. Truncations reduced fusion proportionately to the length of the remaining gH_CT_ to the point of abolishing it altogether (Fig. 5D).

**Fig 6:**
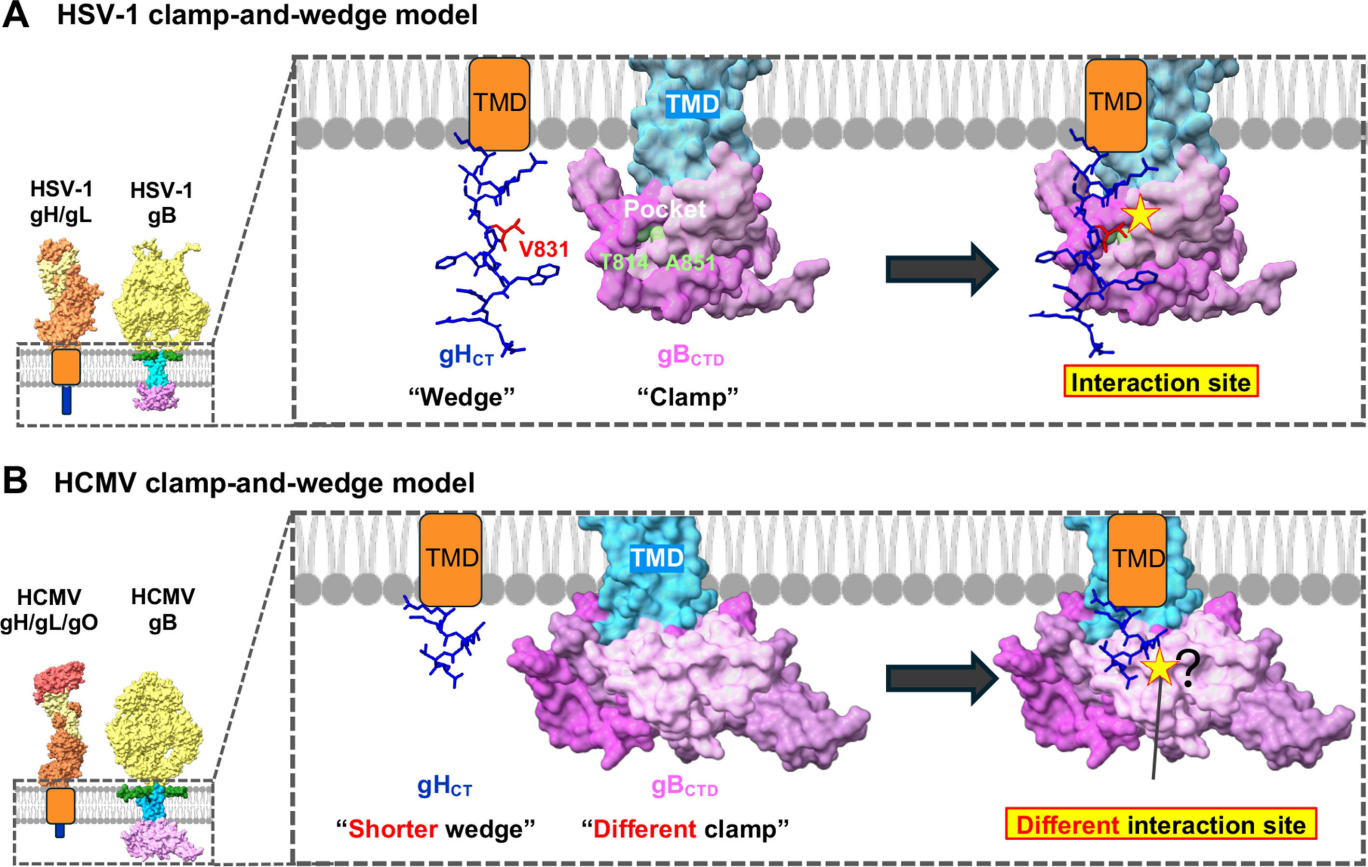
Proposed herpesviral fusion activation mechanism. (**A**) A previously proposed model for HSV-1 fusion triggering with gB_CTD_-gH_CT_ interaction site. In HSV-1, gH_CT_ acts as a wedge and disrupts the inhibitory clamp formed by gB_CTD_ by inserting gH V831 (red) into the gB pocket (T814 and A851, light green). Clamp-and-wedge interaction triggers fusogenic refolding of gB from the prefusion into the postfusion conformation. Structures were rendered in ChimeraX using HSV-1 gH/gL ectodomain (PDB ID 3M1C), HSV-1 gB ectodomain (prefusion) (PDB ID 6Z9M), and HSV-1 gB MPR-TMD-CTD (PDB ID 5V2S). Residues of HSV-1 gH_CT_ were presented using the AlphaFold-predicted gH/gL complex. (**B**) A model of HCMV fusion triggering based on this study. In HCMV, gH_CT_ and gB_CTD_ also act as a wedge and a clamp, respectively. However, HCMV gH_CT_ interacts with different regions of the gB_CTD_, possibly due to different structures of the gB_CTD_ clamp and the shorter gH_CT_ wedge. Structures were rendered in ChimeraX using HCMV gH/gL/gO ectodomain (PDB ID 7LBE), HCMV gB ectodomain (prefusion) (PDB ID 7KDP), and HCMV gB MPR-TMD-CTD (homology modeling). Residues of HCMV gH_CT_ were presented using the AlphaFold-predicted gH/gL/gO complex.

While both the gB_CTD_ and the gH_CT_ are indispensable for HCMV-glycoprotein-induced fusion, our mutational analysis suggests that the gB_CTD_ structure and its interactions with the gH_CT_ differ from their HSV-1 counterparts. First, mutations targeting conserved residues at putative protein/membrane or trimeric interfaces in HCMV gB_CTD_ were either hypo-fusogenic or low-surface level (Fig. 3D and G) instead of hyper-fusogenic as in HSV-1 gB_CTD_. Moreover, mutations targeting the putative surface pocket in HCMV gB_CTD_ were low-surface level (Fig. 4H) instead of hypo-fusogenic or hyper-fusogenic as in HSV-1 gB_CTD_. The most likely explanation for this discrepancy is that the point mutations, which were designed based on the homology-based structural model (Fig. 3B and 4B) and Alphafold-predicted model (Fig. 4C), disrupt the actual HCMV gB_CTD_ structure because it is different from the model. This also suggests that HCMV gB_CTD_ and gH_CT_ interact differently from what has been proposed for HSV-1. In HSV-1, the gH_CT_ acts as a “wedge” that disrupts the gB_CTD_ clamp by inserting residue V831 into the surface pocket (13). Our mutational analysis suggests that this surface pocket is not conserved in the HCMV gB_CTD_. In addition, the 6-residue gH_CT_ lacks the counterpart of V831, which is the 7th residue in HSV-1 gH_CT_. We hypothesize that while in HCMV, the gB_CTD_ may function as an inhibitory clamp and gH_CT_ as an activating wedge, their structures and interactions differ from HSV-1 (Fig. 6). Our results implicate potential mechanistic differences in fusion regulation between herpesvirus subfamilies. Future studies will resolve the structure of the HCMV gB_CTD_ and visualize gB_CTD_/gH_CT_ interactions in HCMV and HSV-1.

Investigation of the fusion phenotypes reported here was made possible by the robust quantitative cell-cell fusion assay. HCMV-induced membrane fusion has traditionally been measured by visualizing syncytia, which is a labor-intensive, semi-quantitative, endpoint method. To overcome these drawbacks, a kinetic SLA used to measure cell-cell fusion induced by HSV-1 glycoproteins was recently adapted to HCMV glycoproteins with recombinant adenoviruses (9). Here, we established an alternative, plasmid-transfection-based SLA and, for the first time, assessed membrane fusion mediated by HCMV glycoproteins from two genetically distinct TR and AD169 strains. By optimizing transfection conditions, we achieved robust signals (200,000–400,000 RLUs over 20 hours) (Fig. 1B and C), representing a fivefold increase in signal intensity over the adenoviral-based HCMV SLA. This enhanced sensitivity enabled accurate analysis of fusion kinetics, revealing that HCMV glycoproteins initiate the fusion approximately 4–6 times later and at slower rates than HSV-1 glycoproteins (Fig. S1E through G). These observations are consistent with the prolonged replication cycle of HCMV, which is 4–6 times longer than that of HSV-1 (43). One limitation of our study is the exclusive use of ARPE-19 cells and mutational analysis in the HCMV TR strain background. In addition, while AD169 and TR have distinct genotypes of gB, gH, and gO glycoproteins, the two strains do not capture full allelic diversity observed across clinical isolates, with nine gB (48), at least two gH (reviewed in reference [60]), and eight gO genotypes (47) currently recognized. Therefore, adapting the HCMV SLA assay to diverse cell types and additional HCMV strains will be important for evaluating the relevance of the proposed fusion mechanism more broadly.

## MATERIALS AND METHODS

### Cells and plasmids

Human ARPE-19 cells (arising retinal pigment epithelial cell line, ATCC CRL-2302) were cultured in Dulbecco’s Modified Eagle Medium/Nutrient Mixture F-12 (DMEM/F-12; Thermo Scientific) containing L-glutamine, HEPES, and Phenol Red and supplemented with 10% heat-inactivated fetal bovine serum (HI-FBS; R&D systems) and 1× penicillin-streptomycin (pen-strep) solution (Corning). Chinese hamster ovary (CHO) cells (a gift from Dr. John M. Coffin, Tufts University) were grown in Ham’s F12 medium (Corning) supplemented with 10% HI-FBS and 1× pen-strep solution. Both ARPE-19 and CHO cells were incubated at 37°C in the presence of 5% CO_2_, detached with trypsin, and subcultured 2–3 times per week.

Plasmids encoding codon-optimized, full-length HCMV (strain TR, Genbank: KF021605.1) genes for gB, gH, gL, and gO in a pDC316(io) vector background were a gift from Dr. Brent Ryckman, University of Montana. These plasmids were derived from replication-defective (E1-negative) adenovirus (Ad) vectors containing the packaging signal, a murine CMV promoter, and SV40 poly A sequences followed by a loxP site (61). Plasmid encoding mutant human PDGFRα^V242K*^, which contains a deletion of residues 1–23 and a V242K substitution within human PDGFRα isoform 1 (GenBank NM_006206.4) in a pDC316(io) vector background was a gift from Dr. Brent Ryckman, University of Montana. PDGFRα^V242K*^ has a wild-type affinity for HCMV gH/gL/gO but does not bind its native ligand PDGF (49), which increases its surface levels due to blocked internalization in the presence of PDGF.

Plasmids RLuc1-7 and RLuc8-11 (carrying the *Renilla* split luciferase genes) (40) were gifts from Dr. Zene Matsuda (University of Tokyo). Plasmid pBG38 carrying the human nectin-1 gene (62) was a gift from Drs. Gary H. Cohen and Roselyn J. Eisenberg (University of Pennsylvania). pPEP98, pPEP99, pPEP100, and pPEP101 encode the full-length HSV-1 (strain KOS, Genbank: JQ780693.1) genes for gB, gD, gH, and gL, respectively, in a pCAGGS vector background and were gifts from Dr. Patricia G. Spear (Northwestern University). Plasmid pJLS11 (encoding HSV-1 gB868 mutant) (32) was previously generated in our laboratory.

### Antibodies

Mouse anti-HCMV-gH monoclonal antibody 14-4b and mouse anti-HCMV-gB monoclonal antibody 27-156 were gifts from Dr. William J. Britt (University of Alabama). Human monoclonal anti-HCMV-gB antibody 1G2 (IgG3) was produced by Genscript. Rabbit polyclonal anti-HSV-gB antibody R69 was a gift from Dr. G. H. Cohen and R. J. Eisenberg (University of Pennsylvania).

### gB_CTD_ mutagenesis

Truncations and single-point mutations in the cytoplasmic domain of the full-length HCMV (strain TR) gB gene were generated in pDC316(io)::HCMV TR gB background by using Phusion HF PCR kit (cat# M0530L) and either Gibson assembly master mix (cat# E2611L) or Quick-Change method. For all gB_CTD_ truncation mutations (gB895, gB884, gB868, gB856, and gB782), two stop codons were introduced after the amino acid of interest by PCR and assembled using the Gibson Assembly method. For gB_CTD_ single-point mutations, 1–3 nucleotide substitutions were introduced by PCR and cloned using either Gibson Assembly method (gB V792L, L851V, L851F, V854A, and V854F) or Quick-Change mutagenesis (gB R788W, P783A, L856A, A858V, R861H, and R861C). Primers used in gB_CTD_ mutagenesis are listed in Table S1. All constructs were confirmed by plasmid sequencing. The resulting plasmids were pLL7 (gB895), pLL8 (gB884), pLL9 (gB868), pLL10 (gB856), pLL11 (gB782), pLL12 (V792L), pLL13 (L851V), pLL14 (L851F), pLL15 (V854A), pLL17 (V854F), pLL25 (R778W), pLL26 (P783A), pLL28 (L856A), pLL29 (A858V), pLL30 (R861H), and pLL31 (R861C).

### gH_CT_ mutagenesis

Truncations in the cytoplasmic tail of the full-length HCMV (strain TR) gH gene were generated in pDC316(io)::HCMV TR gH background by using Phusion HF PCR kit (cat# M0530L) and either Gibson assembly master mix (cat# E2611L) or Quick-Change method. For gH_CT_ truncations, two stop codons were introduced after the amino acid of interest by PCR and cloned using either the Gibson Assembly method (gH737, gH740) or the Quick-Change mutagenesis (gH738, gH739, gH741, and gH742). Primers used in gH_CT_ mutagenesis are listed in Table S2. All constructs were confirmed by plasmid sequencing. The resulting plasmids were pLL19 (gH737), pLL20 (gH738) pLL21 (gH739), pLL22 (gH740), pLL23 (gH741), and pLL24 (gH742).

### Cell-cell fusion assay

Cell-cell fusion mediated by HSV-1 glycoproteins was measured using a SLA following the published protocol (13) with modifications, such as single use of plasmids and EnduRen substrate aliquots, use of a plate reader with proper CO2 level, and seeding cells with proper confluency. Cell-cell fusion mediated by HCMV glycoproteins was measured using a SLA adapted from reference (13). ARPE-19 cells were seeded into 3 wells per condition in a 96-well plate at 10,000 cells per well with 100 µL volume for effector cells and in 6-well plates at 200,000 cells per well with 2 mL volume for target cells.

The next day, when cell confluency is 60%–80%, a total of 100 ng DNA per well was transfected with 30 ng of gB (pDC316(io)::HCMV TR gB or gB_CTD_ mutants), 10 ng of gH (pDC316(io):: HCMV TR gH or gH_CT_ mutants), 10 ng of gL (pDC316(io)::HCMV TR gL), 5 ng of gO (pDC316(io)::HCMV TR gO), 10 ng of split luciferase (pCAGGS::RLuc1-7), and 35 ng of pCAGGS in effector cells using jetPRIME transfection reagent (0.3 µL per well in 10 µL jetPRIME buffer, cat# 89129-924). Target cells were transected with plasmids of a total 2 µg DNA per well with 1 µg of the mutated receptor PDGFRα^V242K*^ (d 1-23 AA, V242K) (pDC316(io)::PDGFRα^V242K*^) and 1 µg of the complementary part of split luciferase (pCAGGS::RLuc8-11) using jetPRIME transfection reagent (4 µg per well in 200 µL jetPRIME buffer) in a dropwise manner. In addition, “No gps” and “No PDGFRɑ,” which stand for deletion of all glycoproteins and PDGFRɑ^V242K*^, respectively, were included as negative controls.

On day 3, at 24 hours post-transfection, the medium of effector cells was replaced with 50 µL per well of fusion medium (DMEM/F-12 with 10% FBS, 1× Pen/Strep, 50 mM HEPES), containing 1:500 EnduRen live cell substrate (Promega, cat# E6482) added. After target cells were detached using 1 mL of Versene (Fisher Scientific, cat# 226-126) per well for 25 min, they were collected, centrifuged, and resuspended in fusion medium of 500 µL per well. Next, 50 µL of resuspended target cells was added to each 96-well of effector cells, and the plate was immediately placed in a BioTek plate reader at 37°C with 5% CO2. Luminescence was recorded every 2 min for 20 hours. Luminescence values were then averaged for the three wells in each condition, normalized to the WT signal at 20 hours, and expressed as a percentage of late fusion extent. For the initiation of fusion, the first time point where luminescence reached a value twofold above the “No gps” control was monitored. Data presented represent the mean with standard error of the mean (SEM) from at least three biological replicates unless otherwise noted.

### Flow cytometry

Cell surface expression of gB_CTD_ and gH_CT_ mutants was measured using flow cytometry. ARPE-19 cells were seeded at 2.5 × 10^5^ cells per well in six-well plates (2 mL per well). The next day, ARPE-19 cells were transfected with a total of 2 µg of DNA using 4 µg per well of jetPRIME transfection reagent in 200 µL jetPRIME buffer (VWR, cat# 89129-924). For measurement of surface expressions of gB_CTD_ mutants, 2 µg of WT gB or gB_CTD_ mutants were transfected per well. For measurement of surface expressions of gH_CT_ truncation mutants, 2 µg of gB, gH, gL, and gO (3:1:1:0.5) were transfected per well. Two wells were left for mock and empty vector (pCAGGS) transfections as negative controls.

On day 3, at 24 hours post-transfection, cells were incubated with 1 mL of Versene per well for 25 min at 37°C, collected in ice-cold FACS medium (PBS with 3% FBS), and washed with FACS media. For gB detection, additionally, cells were blocked with 100 µL of human Fc Block diluted 1:50 in PBS (BD, cat#564220) for 30 minutes at 4°C to prevent non-specific binding of anti-HCMV-gB antibody to human Fc receptor. For primary antibody incubation, cells were incubated with human monoclonal anti-HCMV-gB IgG 1G2 (1:1,000, 100 µL per well) or mouse monoclonal anti-HCMV-gH IgG 14-4b (1:200, 100 µL per well) for 1 hour at 4°C. Cells were then fixed with 4% paraformaldehyde (PFA) in PBS for 20 min at room temperature, washed three times with FACS media, and incubated with Alexa-488-conjugated secondary antibodies (1:250, 100 µL per well) (Alexa488-conjugated goat anti-human IgG for gB and Alexa488-conjugated goat anti-mouse IgG for gH) for 1 hour at 4°C in the dark. Cells were washed three times before resuspending in 250 µL of FACS media. gB- and gH-positive cells were measured by the fluorescence-activated cell sorting method using the pCAGGS-transfected cells with a cutoff of around 2% Alexa488-positive cells to capture most true positives while minimizing false positives. Flow cytometry scatter plots and histograms were generated by plotting single cells and cell count over fluorescence intensity, respectively.

HSV-1 gB surface expressions were measured following the above protocol with modifications. CHO cells were transfected with 2 µg of HSV-1 gB and rabbit polyclonal anti-HSV-gB IgG R69 (1:500, 100 µL per well) and FITC-conjugated goat anti-rabbit IgG (1:250, 100 µL per well) were used for primary and secondary antibodies, respectively.

### Western blots

Total cellular expressions of gB_CTD_ truncation and point mutation constructs were tested using Western blotting. ARPE-19 cells were seeded at 2.5 × 10^5^ cells in 2 mL per well in a 6-well plate. The next day, cells were transfected with 2 µg of WT gB or gB_CTD_ mutant plasmids per well using 4 µg per well of jetPRIME transfection reagent in 200 µL jetPRIME buffer (VWR, cat# 89129-924). On day 3, at 24 hours post-transfection, cells were washed once with ice-cold PBS and collected using a cell scraper (Celltreat, cat# 229310) in a 100 µL of ice-cold radioimmunoprecipitation assay (RIPA) buffer supplemented with 1× Complete protease inhibitor (Sigma-Aldrich, cat# 05056489001) per well. After a 30 minute incubation on ice, cells were centrifuged, supernatants were collected, and protein concentration of each condition was normalized by mixing with SDS-PAGE loading dye after a BCA assay (Thermo Scientific cat# 23227).

Proteins were denatured by heating at 95°C for 5 minutes, separated by SDS-PAGE at 200 V for 30 minutes, and transferred onto nitrocellulose membranes (GE Healthcare, cat # 10600002) using the Trans-Blot Turbo Transfer System (Bio-Rad) at 25 V for 30 minutes. Membranes were then blocked with 3% BSA (Fisher Scientific cat# BP1600100) in TBST for 1 hour at room temperature with gentle rocking. For antibody staining, strips of membranes were incubated overnight at 4°C with the anti-HCMV-gB IgG 27-156 (mAb, mouse IgG2b, 1:1,000) or anti-β-actin IgG (pAb, 1:1,000,000) in 3% BSA/TBST. The next day, membranes were washed three times with TBST and incubated with a fluorescent secondary antibody IRDye 800cw goat anti-mouse IgG (LI-COR Biosciences, cat# 926-32210) or IRDye 800cw goat anti-rabbit IgG (LI-COR Biosciences, cat# 926-32211) (1:5,000) for 1 hour at room temperature with gentle rocking. Membranes were imaged using a Bio-Rad GelDoc system. For quantification of protein expressions, band intensities were adjusted by dividing by the band intensity of actin in ImageJ. Relative cellular expression was measured by normalizing its adjusted band intensity to WT gB intensity.

### Structural analysis

Structural models of the HCMV (TR) gB trimer were generated either using the AlphaFold v.3 online server (https://alphafoldserver.com/) (63) or the SWISS-MODEL homology modeling program (64) using the crystal structure of the HSV-1 gB (PDB: 5V2S) (12) and visualized in ChimeraX (65). The structural models were aligned relative to the membrane using TMHMM 2.0 (https://services.healthtech.dtu.dk/services/TMHMM-2.0). In AlphaFold v.3, the accuracy of prediction for each residue is shown as the local-distance difference test (pLDDT), on a scale from 0 to 100, with higher scores indicating higher quality prediction. In SWISS-MODEL, the accuracy of prediction for each residue is shown as QMEANDisCO (Q mean distance constraint), which represents energies obtained statistically calculated relative to all known experimental 3D structures in the database, on a scale from 0 to 1, with higher scores indicating higher confidence.

### Sequence alignments

gB_CTD_ sequences between HSV-1 (strain KOS) and HCMV (strain TR) were aligned in Clustal Omega and rendered using ESPript 3.0 (https://espript.ibcp.fr) (66). The secondary structures of HSV and HCMV gB_CTD_ were predicted in PSIPRED v.4 (http://bioinf.cs.ucl.ac.uk/psipred) (67). Locations of hyper- and hypo-fusogenic point mutations found in HSV-1 or HSV-2 were indicated above the residue with green and red asterisks, respectively. Locations of point mutations that resulted in limited surface expression in HSV-1 were indicated with purple asterisks. In all sequence alignments, conserved and similar residues of HCMV gB are highlighted in white letter within a red box and in red letter within a blue box, respectively. gH_CT_ sequences from selected herpesviruses, including three alpha-herpesviruses (HSV-1 KOS, HSV-2 333, PRV Kaplan) and eight beta-herpesviruses (HCMV clinical and laboratory adapted strains AD169, JHC, JP, TB40, Toledo, Towne, Merlin, and TR) were aligned using Clustal Omega (68) and rendered with ESPript 3.0 (66) (https://espript.ibcp.fr).

### Statistics

Statistical analysis was performed on the normalized values using Graph-Pad PRISM 9 software. Unpaired t-test with Welch’s correction was used to compare conditions as indicated.

## SUPPLEMENTAL MATERIAL

Supplemental material mbio.01905-25-s0001.pdf Figures S1 to S6; Tables S1 and S2.

ASM does not own the copyrights to Supplemental Material that may be linked to, or accessed through, an article. The authors have granted ASM a non-exclusive, world-wide license to publish the Supplemental Material files. Please contact the corresponding author directly for reuse.
