# Supplementary material for: Mutational dissection of HCMV gB and gH cytoplasmic tails highlights conserved and divergent features of fusion regulation
Source: mBio. 2025 Sep 22;16(11):e01905-25. doi: 10.1128/mbio.01905-25 (PMC12607583; doi:10.1128/mbio.01905-25)
Supplement: Supplemental material — Figures S1 to S6; Tables S1 and S2. [file mbio.01905-25-s0001.pdf]

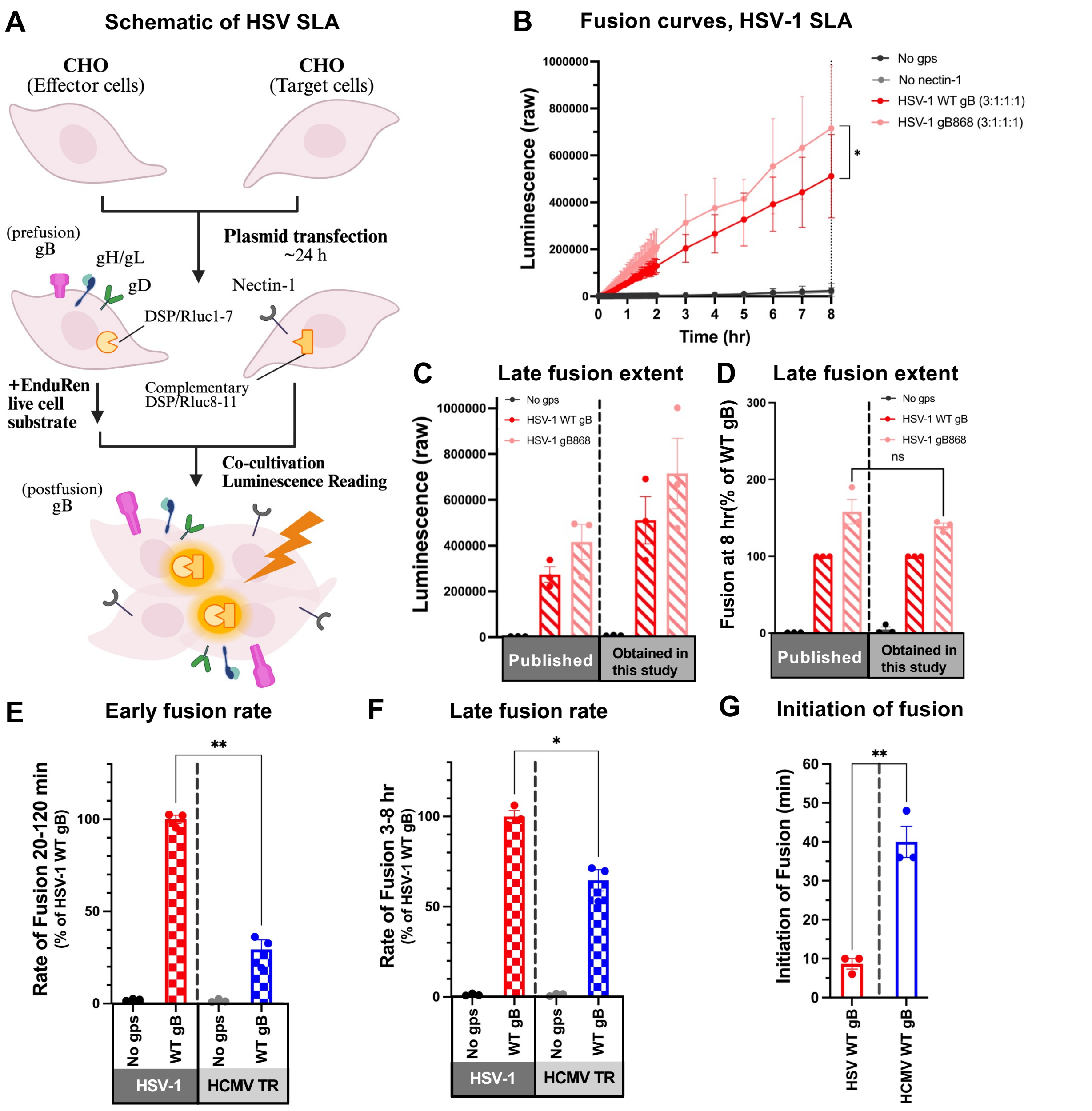

Figure S1. Optimization of a split-luciferase cell-cell fusion assay with HSV-1 entry glycoproteins and comparison of fusion kinetics between HSV-1 and HCMV glycoproteins.

(A) Schematic representation of the experimental setup. CHO cells expressing HSV-1 gB, gH, gL, gD, and a fragment of *Renilla* luciferase (effector cells) are fusing with CHO cells expressing nectin-1 and a complementary fragment of *Renilla* luciferase (target cells). Figure created with Biorender.com. (B) Fusion curve of HSV-1 WT gB and a hyper-fusogenic mutant gB868. Raw luminescence values were plotted over 8 hours post co-cultivation of effector and target cells. Each dot in curves represent a mean raw luminescence value from three biological replicates. Error bars represent SEM. “No gps” and “No nectin-1” served as negative controls. (C-D) Comparison of raw luminescence values (C) and late extent of fusion (D) from the published study (Pataki et al., 2022) and this study. (E-G) Comparison of early fusion rate (E), late fusion rate (F), and initiation of fusion (G) for HSV-1 and HCMV glycoproteins. Early fusion rate was defined as the slope of the fusion curve between 20-120 minutes (HSV-1) or 20-120 minutes (HCMV) post co-culture of effector and target cells. Late fusion rate was defined as the slope of the fusion curve between 3-8 hours (HSV-1) or 3-8 hours (HCMV) post co-culture of effector and target cells. Early and late fusion rates for HCMV were normalized to HSV-1 WT gB. Initiation of fusion was defined as the first time point where luminescence reached the value 2-fold above the “No gps” control. Bars represent the mean values from three biological replicates, each of which is the mean of three technical replicates. Error bars represent SEM. *: $p < 0.05$, **: $p < 0.01$, ***: $p < 0.001$, ****: $p < 0.0001$.

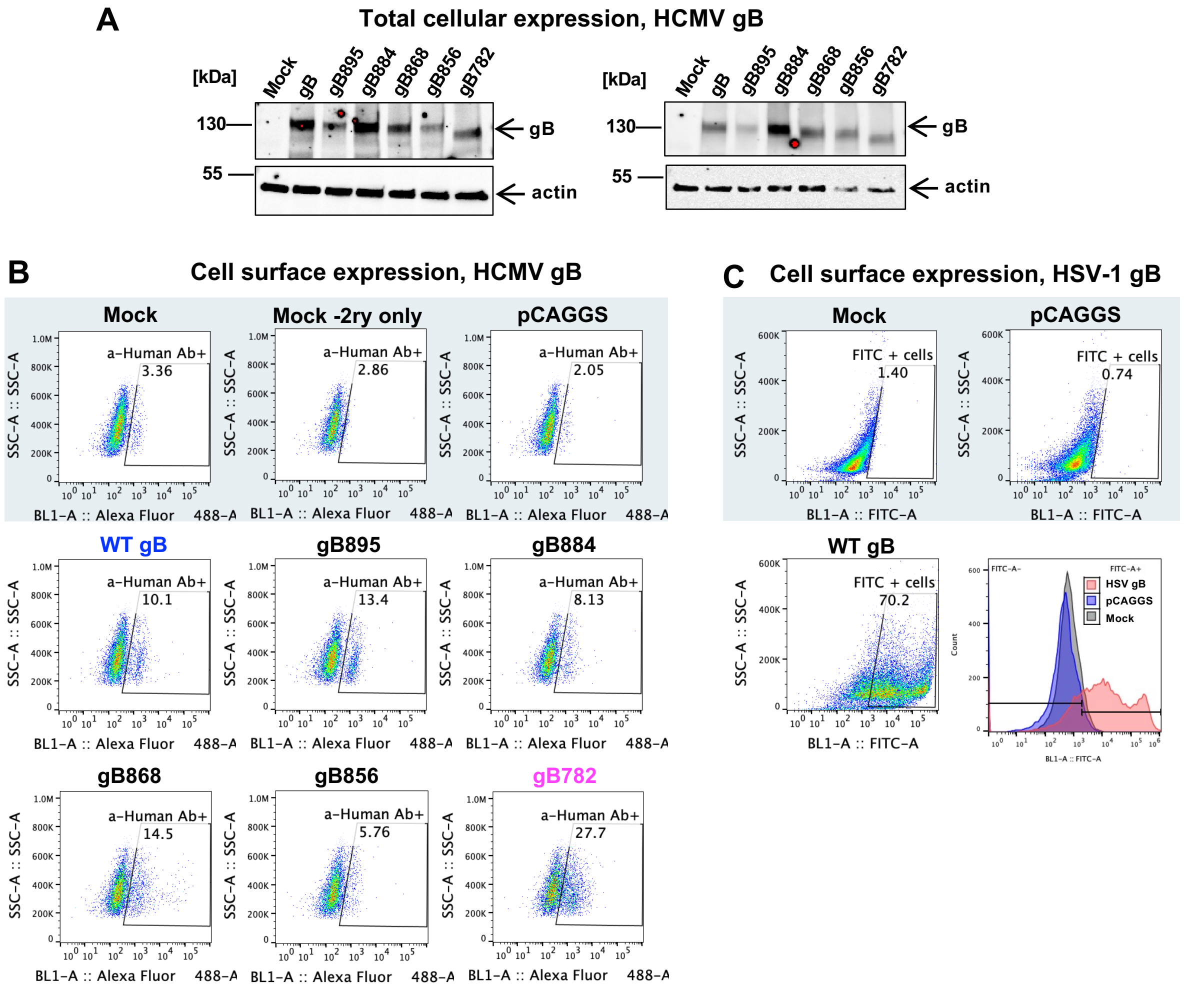

Figure S2. Total cellular expression and cell surface expression of HCMV WT gB and gB$_{CTD}$ truncation mutants. (A) Western blots were probed with an anti-HCMV-gB mouse IgG 27-156 as a primary and IRDye 800cw goat anti-mouse IgG as a secondary antibody. Mock-transfected ARPE-19 cells ("Mock") served as a negative control. Actin served as a loading control. Two representative western blots out of three biological replicates are shown. Quantification is shown in Figure 2G. (B) Cell surface expression levels of HCMV TR WT gB and gB$_{CTD}$ truncation mutants were measured by flow cytometry using anti-HCMV-gB human IgG 1G2 as a primary and Alexa488-conjugated goat anti-human IgG as a secondary antibody. ARPE-19 cells mock transfected ("Mock") or transfected with an empty vector ("pCAGGS") served as negative controls. Representative flow cytometry scatter plots are shown. HCMV WT gB and gB782 are colored in blue and magenta, respectively. Quantification is shown in Figure 2H. (C) Cell surface expression level of HSV-1 KOS WT gB was measured by flow cytometry using anti-HCMV-gB rabbit pAb R69 as a primary and FITC-conjugated anti-rabbit IgG as a secondary antibody. ARPE-19 cells mock transfected ("Mock") or transfected with an empty vector ("pCAGGS") served as negative controls.

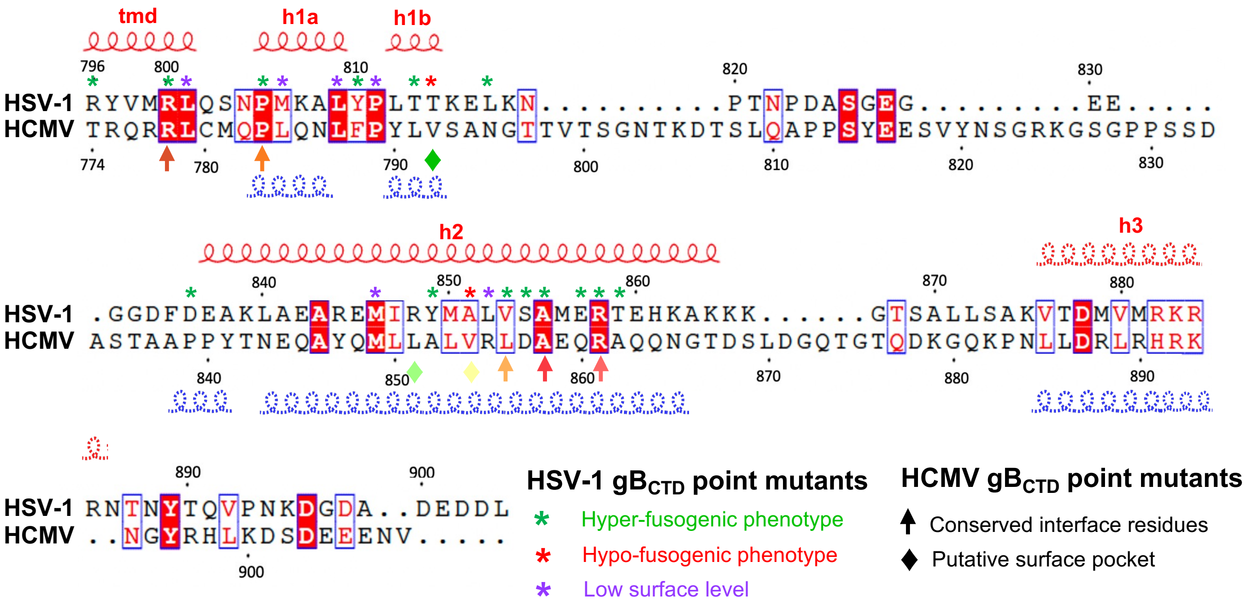

Figure S3. Sequence alignment of gB_{CTD} from HSV-1 KOS and HCMV TR and a comparison of surface pockets in gB_{CTD} in HSV-1 and HCMV. Sequence alignment of the gB_{CTD} from HSV-1 KOS and HCMV TR. Sequence alignment was generated in Clustal Omega and rendered using ESPript 3.0. Identical residues are shown in white on red background, and similar residues are shown in red. Known point mutations in HSV-1 and HSV-2 gB_{CTD} with hyper-fusogenic, hypo-fusogenic, or low surface level phenotypes are marked with green, red, or purple asterisks, respectively. Point mutations in HCMV gB_{CTD} tested in this study are indicated with arrows and diamonds, in colors matching Figures 3 and 4, respectively.

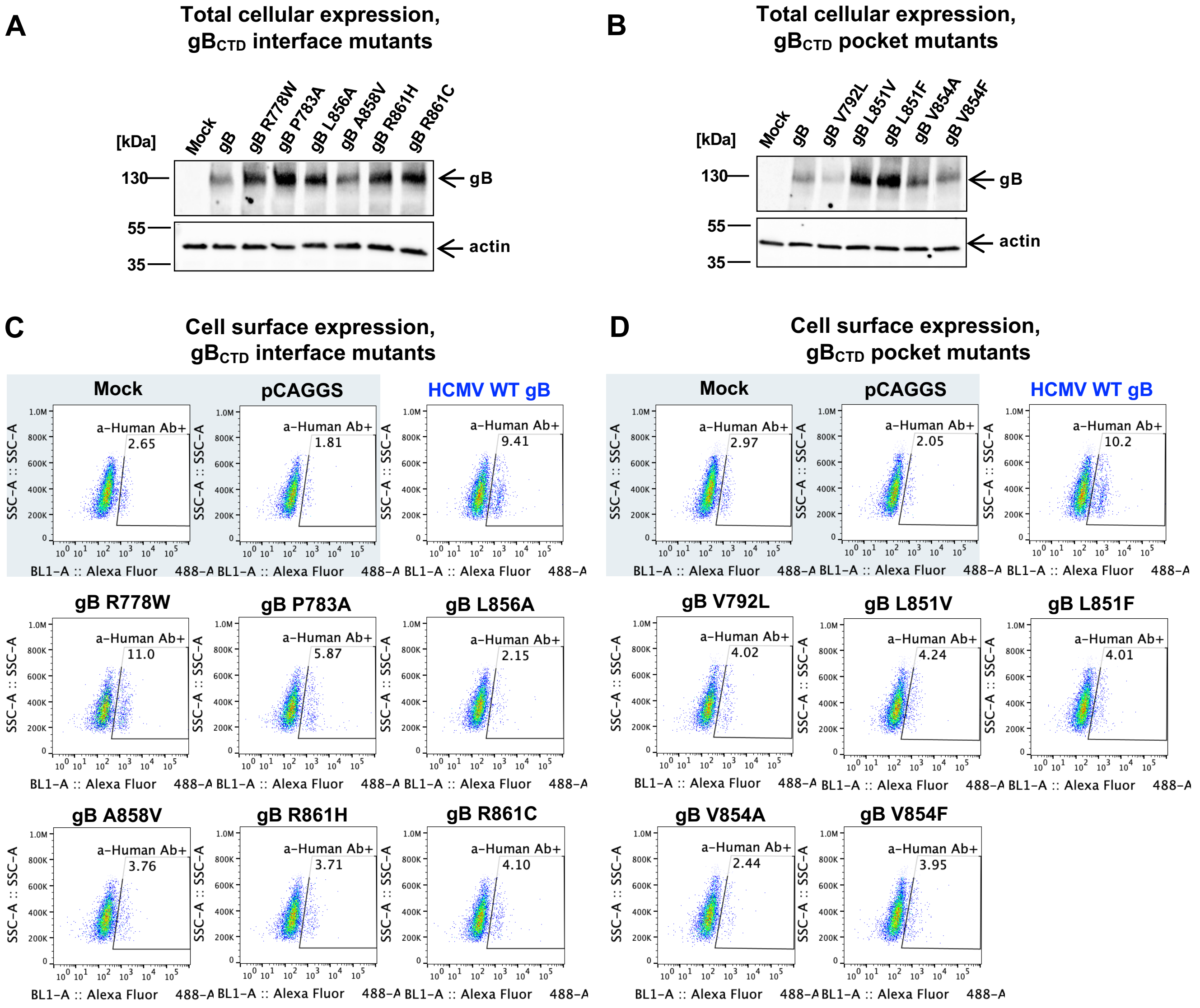

Figure S4. Total cellular expression and cell surface expression of HCMV gB_{CTD} point mutants, interface and pocket. (A-B) Western blots were probed with an anti-HCMV-gB mouse IgG 27-156 as a primary and IRDye 800cw goat anti-mouse IgG as a secondary antibody. Mock-transfected ARPE-19 cells ("Mock") served as a negative control. Actin served as a loading control. One representative western blot out of three biological replicates of gB_{CTD} interface mutants (A) and gB_{CTD} pocket mutants (B) is shown. Quantifications are shown in Figures 3F and 4G. (C-D) Cell surface expression levels of HCMV TR WT gB and gB_{CTD} single-point mutants were measured by flow cytometry using anti-HCMV-gB human IgG 1G2 as a primary and Alexa488-conjugated goat anti-human IgG as a secondary antibody. ARPE-19 cells mock transfected ("Mock") or transfected with an empty vector ("pCAGGS") served as negative controls. Representative flow cytometry scatter plots of gB_{CTD} interface mutants (C) and gB_{CTD} pocket mutants (D) are shown. Quantifications are shown in Figure 3G and 4H. HCMV WT gB is colored in blue.

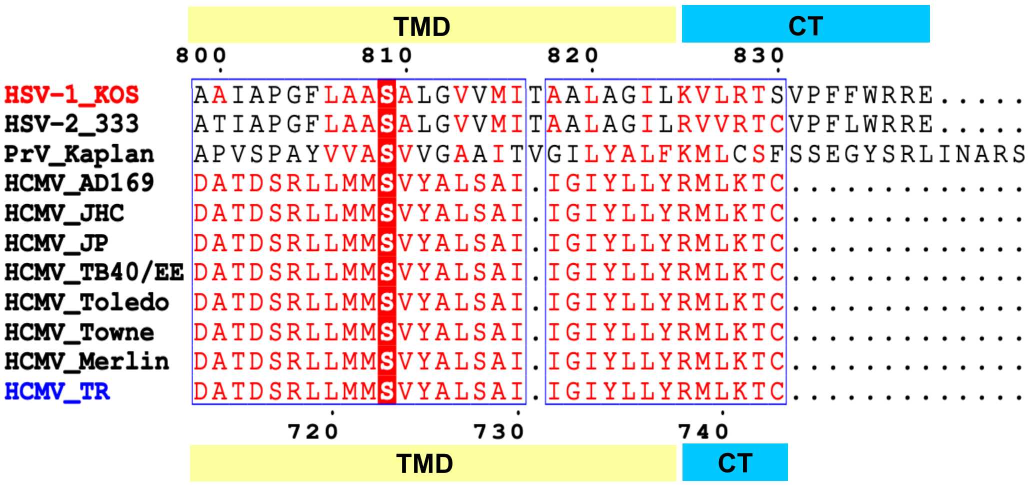

Figure S5. Sequence alignment of gH_{TMD-CT} sequences from HSV-1 KOS, HSV-2 333, PRV Kaplan, and eight HCMV strains: AD169, JHC, JP, TB40, Toledo, Towne, Merlin, and TR. Sequence alignment was generated in Clustal Omega and rendered using ESPript 3.0. Identical residues are shown in white on red background, and similar residues are shown in red.

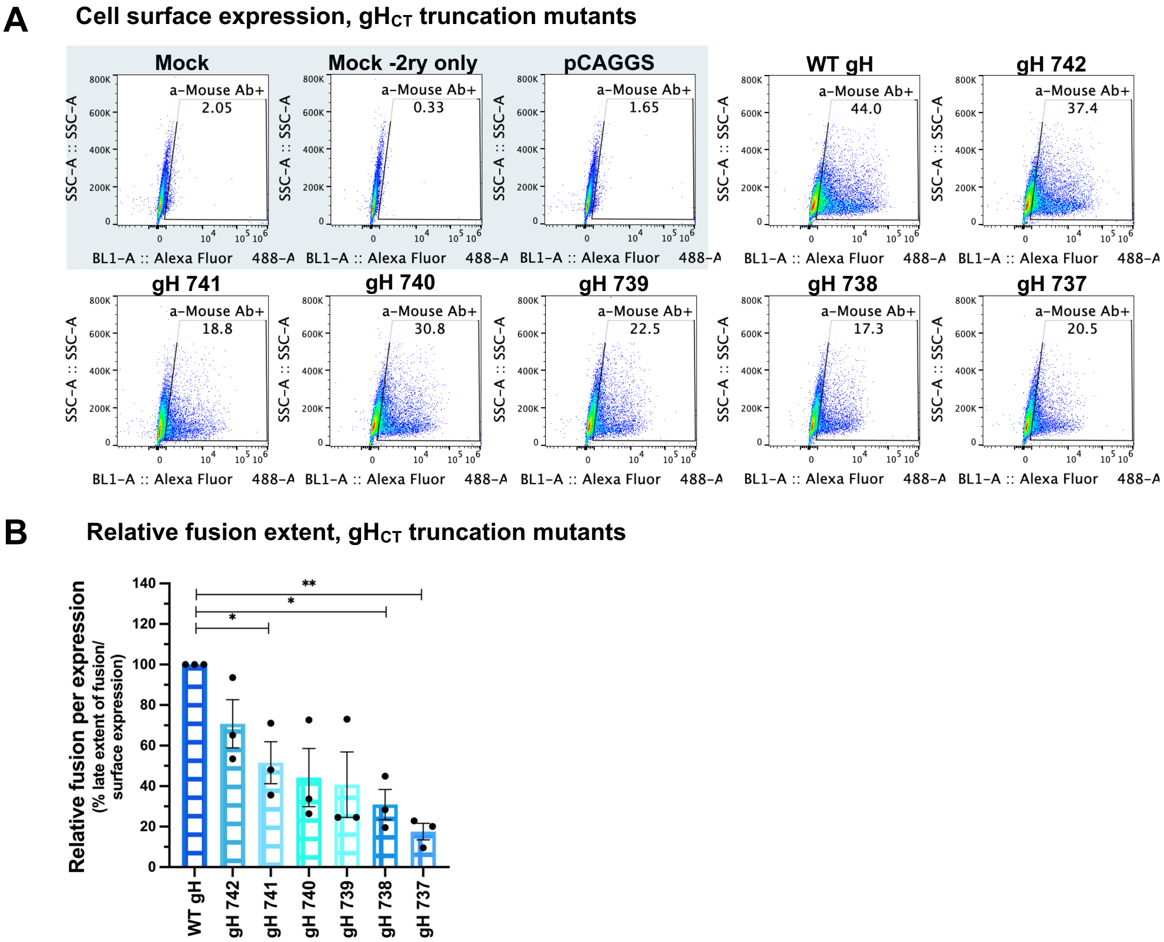

Figure S6. Cell surface expression of HCMV gH_{CT} truncation mutants in ARPE-19 cells. Cell surface expression levels were measured by flow cytometry using anti-HCMV-gH mouse IgG 14-4b as a primary and Alexa488-conjugated goat anti-mouse IgG as a secondary antibody. ARPE-19 cells mock transfected ("Mock") or transfected with an empty vector ("pCAGGS") served as negative controls. (A) Representative flow cytometry scatter plots. (B) Relative fusion extent of gH_{CT} truncation mutants by dividing the late extent of fusion by the surface expression. All normalization was performed based on a set of biological replicates conducted on the same day. Error bars representing SEM. *: $p < 0.05$, **: $p < 0.01$.

gB PCR fragments	**Forward primer**<br>**Reverse primer**
gB895 piece 1	5’- TGT GGC GGA AGT GTG ATG TTG CAA GTG TG - 3’<br>5’ - AGA GTC TTT CAA GTG TC**A T**TA GCC GTT TTT GCG ATG – 3’
gB895 piece 2	5’ - CAT CGC AAA AAC GGC TA**A T**GA CAC TTG AAA GAC TCT – 3’<br>5’ - CAC ACT TGC AAC ATC ACA CTT CCG CCA CA – 3’
gB884 piece 1	5’ - TGT GGC GGA AGT GTG ATG TTG CAA GTG TG – 3’<br>5’ - ATG TCG CAG TCG GTC T**TA** C**TA** GTT GGG CTT CTG TCC – 3’
gB884 piece 2	5’ - GGA CAG AAG CCC AAC **TA**G **TA**A GAC CGA CTG CGA CAT – 3’<br>5’ - CAC ACT TGC AAC ATC ACA CTT CCG CCA CA – 3’
gB868 piece 1	5’ - TGT GGC GGA AGT GTG ATG TTG CAA GTG TG – 3’<br>5’ - GCC AGT CTG TCC GTC C**T**A **TC**A ATC TGT ACC GTT CTG – 3’
gB868 piece 2	5’ - CAG AAC GGT ACA GAT T**GA** T**A**G GAC GGA CAG ACT GGC – 3’<br>5’ - CAC ACT TGC AAC ATC ACA CTT CCG CCA CA – 3’
gB856 piece 1	5’ - TGT GGC GGA AGT GTG ATG TTG CAA GTG TG – 3’<br>5’ - CTC GCT GCT CT**C AC**T **A**CA GAC GGA CCA GGG CCA GAA – 3’
gB856 piece 2	5’ - TTC TGG CCC TGG TCC GTC TG**T** A**GT G**AG AGC AGC GAG – 3’<br>5’ - CAC ACT TGC AAC ATC ACA CTT CCG CCA CA – 3’
gB782 piece 1	5’ - TGT GGC GGA AGT GTG ATG TTG CAA GTG TG – 3’<br>5’ - AGA TAG GGA AAG AGG TTC TGC **TA**C **TA**C TGC ATG CA – 3’
gB782 piece 2	5’ - TGC ATG CAG **TA**G **TA**G CAG AAC CTC TTT CCC TAT CT – 3’<br>5’ - CAC ACT TGC AAC ATC ACA CTT CCG CCA CA – 3’
gB R778W	5’ - CTA TAC TCG ACA GCG G**T**G **G**CT CTG CAT GCA GC – 3’<br>5’ - GCT GCA TGC AGA G**C**C **A**CC GCT GTC GAG TAT AG – 3’
gB P783A	5’ - CGT CTC TGC ATG CAG **G**CG CT**A** CA**A** AAC CTC TTT CCC – 3’<br>5’ - GGG AAA GAG GTT **T**TG **T**AG CG**C** CTG CAT GCA GAG ACG – 3’
gB L856A	5’ - GCC CTG GTC CGT **GCA** GAC GCA GAG CAG – 3’<br>5’ - CTG CTC TGC GTC **TGC** ACG GAC CAG GGC - 3 ‘
gB A858V	5’ - CTG GTC CGT CTG GA**T** G**T**A GAG CAG CGA GCG – 3’<br>5’ - CGC TCG CTG CTC T**A**C **A**TC CAG ACG GAC CAG – 3’
gB R861H	5’ - GAC GCA GAG CAG C**AC** GCG CAG CAG AAC – 3’<br>5’ - GTT CTG CTG CGC **GT**G CTG CTC TGC GTC – 3’
gB R861C	5’ - GAC GCA GAG CAG **T**G**T** GC**C** CAG CAG AAC GGT AC – 3’<br>5’ - GTA CCG TTC TGC TG**G** GC**A** C**A**C TGC TCT GCG TC – 3’
gB V792L piece 1	5’ - TGT GGC GGA AGT GTG ATG TTG CA – 3’<br>5’ - CCG TTG GCG GAC A**G**C AGA TAG GGA AA – 3’
gB V792L piece 2	5’ - TTT CCC TAT CTG **C**TG TCC GCC AAC GG – 3’<br>5’ - TG CAA CAT CAC ACT TCC GCC ACA – 3’
gB L851V piece 1	5’ - TGT GGC GGA AGT GTG ATG TTG CA – 3’<br>5’ - ACG GAC CAG GGC CA**C** AAG CAT CTG – 3’
gB L851V piece 2	5’ - CAG ATG CTT **G**TG GCC CTG GTC CGT – 3’<br>5’ - TG CAA CAT CAC ACT TCC GCC ACA – 3’
gB L851F piece 1	5’ - TGT GGC GGA AGT GTG ATG TTG CA – 3’<br>5’ - ACG GAC CAG GGC **G**A**A** AAG CAT CTG – 3’
gB L851F piece 2	5’ - CAG ATG CTT **T**T**C** GCC CTG GTC CGT - 3’<br>5’ - TG CAA CAT CAC ACT TCC GCC ACA – 3’
gB V854A piece 1	5’ - TGT GGC GGA AGT GTG ATG TTG CA – 3’<br>5’ - GTC CAG ACG **AG**C CAG GGC CAG AA – 3’
gB V854A piece 2	5’ - TTC TGG CCC TGG **CT**C GTC TGG AC – 3’<br>5’ - TG CAA CAT CAC ACT TCC GCC ACA – 3’
gB V854F piece 1	5’ - TGT GGC GGA AGT GTG ATG TTG CA – 3’<br>5’ - GTC CAG ACG GA**A** CAG GGC CAG AA – 3’
gB V854F piece 2	5’ - TTC TGG CCC TG**T** TCC GTC TGG AC – 3’<br>5’ - TG CAA CAT CAC ACT TCC GCC ACA – 3’

Table S1. Primers used for gB_{CTD} mutagenesis. For the Gibson Assembly method, two sets of forward and reverse primers, each 25-45 nucleotides (nts) long, were designed. These primers included at least 10-15 nts of perfect matching sequence on both ends of the desired mutation(s) to amplify two DNA fragments. For the Quick-Change mutagenesis, a single pair of forward and reverse primers was designed to anneal to opposite strands of the plasmids, each 25-45 nucleotides long and containing the desired mutation(s). Mutations are indicated in bold, underlined letters.

gH PCR fragments	Forward primer<br>Reverse primer
gH742	5’ - CGG ATG CTG AAA ACC TG**A** TGA TGA GTC GAC TTC – 3’<br>5’ - GAA GTC GAC TCA TCA **T**CA GGT TTT CAG CAT CCG – 3’
gH741	5’ - CGG ATG CTG AAA **TAG** T**AG** TGA TGA GTC GAC TTC G – 3’<br>5’ - CGA AGT CGA CTC ATC A**CT** A**CT A**TT TCA GCA TCC G – 3’
gH740 piece 1	5’ - TGT GGC GGA AGT GTG ATG TTG CAA GTG TG – 3’<br>5’ - GTC GAC TCA TCA GCA **CTA** TT**A** CAG CAT CCG GTA CAG – 3’
gH740 piece 2	5’ - CTG TAC CGG ATG CTG **T**AA **TAG** TGC TGA TGA GTC GAC – 3’<br>5’ - CAC ACT TGC AAC ATC ACA CTT CCG CCA CA – 3’
gH739	5’ - CTG CTG TAC CGG ATG TA**G** T**AA** ACC TGC TGA TGA GTC – 3’<br>5’ - GAC TCA TCA GCA GGT **TT**A **C**TA CAT CCG GTA CAG CAG - 3’
gH738	5’ - TAC CTG CTG TAC CGG **TA**G **TA**G AAA ACC TGC TGA TGA – 3’<br>5’ - TCA TCA GCA GGT TTT C**TA** C**TA** CCG GTA CAG CAG GTA – 3’
gH737 piece 1	5’ - TGT GGC GGA AGT GTG ATG TTG CAA GTG TG – 3’<br>5’ - TCA GCA GGT TTT CAG C**TA T**C**A** GTA CAG CAG GTA GAT – 3’
gH737 piece 2	5’ - ATC TAC CTG CTG TAC **T**G**A** TAG CTG AAA ACC TGC TGA – 3’<br>5’ - CAC ACT TGC AAC ATC ACA CTT CCG CCA CA – 3’

Table S2. Primers used for gH_{CT} mutagenesis. For the Gibson Assembly method, two sets of forward and reverse primers, each 25-45 nucleotides (nts) long, were designed. These primers included at least 10-15 nts of perfect matching sequence on both ends of the desired mutation(s) to amplify two DNA fragments. For the Quick-Change mutagenesis, a single pair of forward and reverse primers was designed to anneal to opposite strands of the plasmids, each 25-45 nucleotides long and containing the desired mutation(s). Mutations are indicated in bold, underlined letters.
